# Definitive evidence of the presence of 24-methylenecycloartanyl ferulate and 24-methylenecycloartanyl caffeate in barley

**DOI:** 10.1038/s41598-019-48985-6

**Published:** 2019-08-29

**Authors:** Junya Ito, Kazue Sawada, Yusuke Ogura, Fan Xinyi, Halida Rahmania, Tomoyo Mohri, Noriko Kohyama, Eunsang Kwon, Takahiro Eitsuka, Hiroyuki Hashimoto, Shigefumi Kuwahara, Teruo Miyazawa, Kiyotaka Nakagawa

**Affiliations:** 1 Food and Biodynamic Chemistry Laboratory, Graduate School of Agricultural Science, Tohoku University, Sendai, Miyagi 980-8572 Japan; 2 Tsuno Food Industrial CO., LTD., Ito-Gun, Wakayama, 649-7194 Japan; 3 Laboratory of Applied Bioorganic Chemistry, Graduate School of Agricultural Science, Tohoku University, Sendai, Miyagi 980-8572 Japan; 4 Institute of Crop Science, National Agriculture and Food Research Organization, Tsukuba, Ibaraki 305-8518 Japan; 5 Research and Analytical Center for Giant Molecules, Graduate School of Science, Tohoku University, Sendai, Miyagi 980-8578 Japan; 6 Food and Health Science Research Unit, Graduate School of Agricultural Science, Tohoku University, Sendai, Miyagi 980-8572 Japan; 7 Food and Biotechnology Innovation Project, New Industry Creation Hatchery Center (NICHe), Tohoku University, Sendai, Miyagi 980-8579 Japan

**Keywords:** Nutrition, Drug development

## Abstract

γ-Oryzanol (OZ), which has a lot of beneficial effects, is a mixture of ferulic acid esters of triterpene alcohols (i.e., triterpene alcohol type of OZ (TTA-OZ)) and ferulic acid esters of plant sterols (i.e., plant sterol type of OZ (PS-OZ)). Although it has been reported that OZ is found in several kinds of cereal typified by rice, TTA-OZ (e.g., 24-methylenecycloartanyl ferulate (24MCA-FA)) has been believed to be characteristic to rice and has not been found in other cereals. In this study, we isolated a compound considered as a TTA-OZ (i.e., 24MCA-FA) from barley and determined the chemical structure using by HPLC-UV-MS, high-resolution MS, and NMR. Based on these results, we proved for the first time that barley certainly contains 24MCA-FA (i.e., TTA-OZ). During the isolation and purification of 24MCA-FA from barley, we found the prospect that a compound with similar properties to OZ (compound-X) might exist. To confirm this finding, the compound-X was also isolated, determined the chemical structure, and identified as a caffeic acid ester of 24-methylenecycloartanol (24MCA-CA), which has rarely been reported before. We also quantified these compounds in various species of barley cultivars and found for the first time the existence of 24MCA-FA and 24MCA-CA in various barley. Through these findings, it opens the possibility to use barley as a new source of 24MCA-FA and 24MCA-CA.

## Introduction

**Figure 1:**
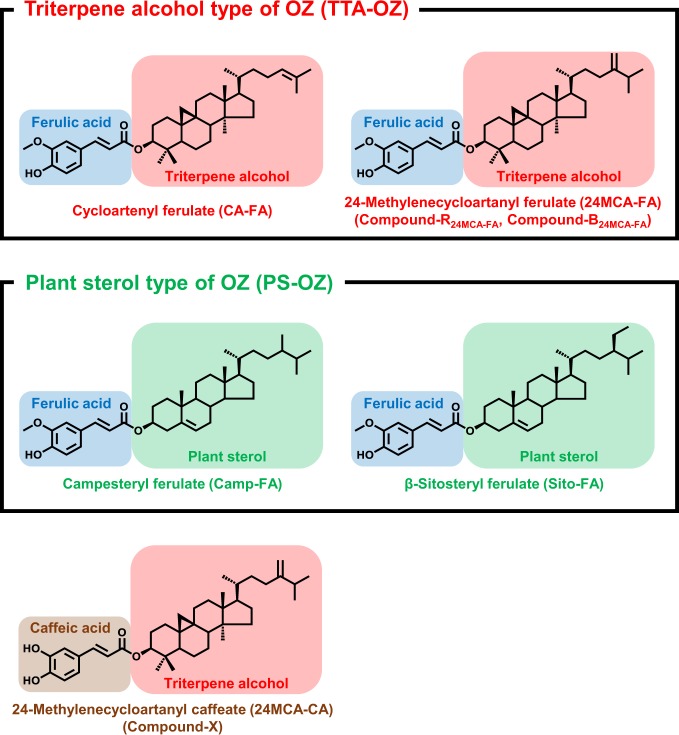
Chemical structures of OZ molecular species (cycloartenyl ferulate, 24-methylenecycloartanyl ferulate, campesteryl ferulate, and β-sitosteryl ferulate) and 24-methylenecycloartanyl caffeate.

γ-Oryzanol (OZ) was first identified from rice bran about 60 years ago. Since a lot of beneficial effects of OZ have been reported (e.g., lipid-lowering, anti-oxidative, antidiabetic, neuroprotective, anticarcinogenic, and immunomodulatory effects)^1–8^, the study about finding the new source of OZ has also been explored. OZ has been reported to be found not only in rice but also in several cereals like wheat, barley, and corn^9,10^. Based on the chemical structure, OZ is divided into two groups; the first group is a ferulic acid esters of triterpene alcohols (i.e., triterpene alcohol type of OZ (TTA-OZ)), typified by cycloartenyl ferulate (CA-FA) and 24-methylenecycloartanyl ferulate (24MCA-FA); and the second one is a ferulic acid esters of plant sterols (i.e., plant sterol type of OZ (PS-OZ)) as represented by campesteryl ferulate (Camp-FA) and β-sitosteryl ferulate (Sito-FA)^11^ (Fig. 1). TTA-OZ has been believed to be characteristic to rice and has not been found in other cereals. In one example, Jiang *et al*. reported that the concentration of TTA-OZ in rice was about 2.5 mg/g total lipids, while in the other cereals (e.g., wheat and barley), the concentration was below the detection limit^9^.

However, in more recent studies, there were some reports suggesting the presence of TTA-OZ in another grain than rice. Lee *et al*. reported the presence of 24MCA-FA in barley after comparison with a 4-component OZ mixture standard by using HPLC-UV^12^. This finding was also strengthened by Tsuzuki *et al*. that has detected the presence of 24MCA-FA in barley through its molecular weight by using an atmospheric pressure chemical ionization method combined with mass spectrometry^13^. However, no studies have conducted the isolation of 24MCA-FA from barley and the determination of its chemical structure using by high-resolution (HR-) MS, and NMR, moreover, the quantification of 24MCA-FA in various barley cultivars.

In this study, we evaluated more about the presence of 24MCA-FA in barley and determined its chemical structure by isolating the compound considered as 24MCA-FA (hereafter referred to as compound-B_24MCA-FA_) and analyzing it by using HPLC-UV-MS, HR-MS, and NMR. During the isolation and purification of compound-B_24MCA-FA_ from barley, we found the prospect that compound with similar properties to OZ (hereafter referred to as compound-X) might exist. To confirm this finding, we also isolated and analyzed compound-X by using HPLC-UV-MS, HR-MS, and NMR. This study has also carried out the quantification of compound-B_24MCA-FA_ and compound-X in various species of barley with different characteristics type.

## Materials and Methods

### Materials

OZ mixture prepared by extracting rice bran was obtained from Tsuno Food Industrial Co., Ltd. (Wakayama, Japan). Barley bran was obtained from Takabatake Seibaku Co., Ltd. (Kagawa, Japan). Grains of barley (*Hordeum vulgare* L.) cv. ‘Mikamo Golden’, ‘Nishinohoshi’, ‘Kashima Goal’, ‘Minorimugi’, ‘Kirarimochi’, ‘Beau Fiber’, ‘Ichibanboshi’, and ‘Sanukihadaka’ were harvested in an experimental field of the National Agriculture and Food Research Organization at Tsukuba in 2017. Whole barley grains were pearled into 55% yield in hulled barley or 60% yield in hull-less barley with a TM-05C test mill (Satake Co., Ltd., Hiroshima, Japan), in order to separate pearled grains and bran. All other reagents were of the highest grade available.

### Isolation of 24MCA-FA standard from rice bran

Standard of 24MCA-FA was isolated from commercial OZ mixture prepared by extracting rice bran. In brief, target compound considered as 24MCA-FA (hereafter referred to as compound-R_24MCA-FA_) was obtained from 10 g of OZ mixture, mainly containing CA-FA, 24MCA-FA, Camp-FA, and Sito-FA, by using crystallization technique with ethyl acetate and ethanol. After four times of crystallization, the crude crystals of compound-R_24MCA-FA_ were obtained and 150 mg of the crude crystals were subjected to flash-chromatography with ODS column (YMC-DispoPackAT ODS, 12 g; YMC CO., Ltd., Kyoto, Japan) and a binary gradient (solvent A (acetonitrile) and solvent B (methanol)). The gradient profile was described as follow: 0–10 min, 0% B; 10–50 min, 0–100% B linear. After the flash-chromatography, 110 mg of compound-R_24MCA-FA_ was obtained. To determine the chemical structure, obtained compound-R_24MCA-FA_ was subjected to HR-ESI-MS (micrOTOF-Q II mass spectrometer, Bruker Daltonik, Bremen, Germany), HR-FAB-MS (JMS-700, JEOL Ltd., Tokyo, Japan) with *m*-nitrobenzyl alcohol as a matrix, ^1^H, ^13^C, and 2D NMR (correlation spectroscopy (COSY), heteronuclear single-quantum correlation spectroscopy (HSQC), heteronuclear multiple-bond correlation spectroscopy (HMBC)) (Varian 600TT, Palo Alto, CA, U.S.A.) at 600 MHz using CDCl_3_ as a solvent, and X-ray crystal structural analysis (R-AXIS RAPID, Rigaku, Tokyo, Japan).

### Determination of chemical structure of the compound-B_24MCA-FA_ and the compound-X from barley bran

The compound considered as 24MCA-FA (hereafter referred to as compound-B_24MCA-FA_) was isolated from barley bran (100 g). Total lipid fraction (3.8 g) was extracted by using Soxhlet-extraction with hexane. Obtained total lipid fraction was subjected to liquid–liquid extraction with acetonitrile, methanol, and 2-propanol. The crude fraction (1.1 g) containing compound-B_24MCA-FA_ was purified by using flash-chromatography with silica column (YMC-DispoPackAT Silica-25, 120 g; YMC CO., LTD.) and amino column (YMC-DispoPackAT NH2, 120 g; YMC CO., LTD.), and approximately 0.86 mg of compound-B_24MCA-FA_ was obtained. Another OZ molecular species (hereafter referred to as compound-X) was isolated from barley bran (300 g). First, total lipid was extracted by using Soxhlet extraction with hexane. The crude compound-X fraction (8.0 g) was obtained from total lipid fraction, by using liquid–liquid extraction with acetonitrile, methanol, and 2-propanol. After liquid–liquid extraction, the crude fraction of compound-X was subjected to flash-chromatography with silica column (YMC-DispoPackAT Silica-25, 120 g; YMC CO., LTD.), amino column (YMC-DispoPackAT NH2, 12 g; YMC CO., LTD.), and ODS column (YMC-DispoPackAT ODS, 12 g; YMC CO., LTD.). From that process, 18 mg of the compound-X was obtained, then it was purified by HPLC-UV with ODS column (Cadenza CL-C18, 3 µm, 10 ID × 250 mm; Imtakt Co., Ltd., Kyoto, Japan). Finally, the compound-X (3.7 mg) was successfully isolated. In order to determine the chemical structure, the pure compound-B_24MCA-FA_ and compound-X were subjected to HR-ESI-MS, HR-FAB-MS with *m*-nitrobenzyl alcohol as a matrix, ^1^H, ^13^C, and 2D NMR (COSY, HSQC, HMBC) at 600 MHz using CDCl_3_ as a solvent.

### Quantification of the compound-B_24MCA-FA_ and the compound-X in barley using HPLC-MS/MS

Whole and pearled barley grains from eight cultivars (i.e., total sixteen samples (Supplementary Information 1)) were pulverized by a mill (Analytical mill A10, IKA, Osaka, Japan) into powder. Extraction of OZ was carried out based on our previous method^11,14,15^ with slight modifications. Briefly, 4.5 mL of 1.5% KCl aqueous solution was added to the barley powder (1 g). Total lipids were extracted from the powder solution using 18 mL of a chloroform–methanol solution (2:1, v/v) with 0.002% butylated hydroxytoluene. The extract was partitioned into two layers by centrifugation (1,200 g, 20 min, −20 °C): the upper layer is the polar one (containing methanol–water), and the lower layer is the non-polar one (the organic layer, containing chloroform). The lower chloroform layer (lipid fraction) was collected. The remaining aqueous layer was re-exctracted by adding 10.5 mL of a chloroform–methanol solution (10:1, v/v) and centrifuging under the same condition (1,200 g, 20 min, −20 °C). The combined lipid fraction was evaporated under nitrogen gas. The dried extract (5 mg) was re-dissolved in 900 µL hexane–chloroform (9:1, v/v), and the solution was loaded onto a Strata SI-1 Silica cartridge (Phenomenex Inc., California, U.S.A.) equilibrated with hexane–chloroform (9:1, v/v). The cartridge was rinsed with 1.4 mL hexane–chloroform (9:1, v/v) and OZ was eluted with 1.4 mL hexane–2-propanol (7:3, v/v). The eluent was evaporated, and the residue was dissolved in 600 µL methanol and filtrated by Nanosep with 0.2 µm Bio-Inert (Pall Corporation, New York, U.S.A). A 2 to 10 µL final aliquot was subjected to HPLC-MS/MS analysis.

The HPLC-MS/MS analysis consisted of a liquid chromatography system (Shimadzu, Kyoto, Japan) and a 4000 QTRAP mass spectrometer (SCIEX, Tokyo, Japan). All analytes were detected by the multiple reaction monitoring (MRM)^14^. The MRM ion pairs and analytical conditions were shown in Supplementary Information 2. All analytes were separated using an HPLC column (COSMOSIL 2.5C18-MS-II, 2.5 µm, 2.0 ID × 100 mm; Nacalai Tesque, Inc., Kyoto, Japan) at 40 °C with a flow rate of 0.5 mL/min. Gradient elution was performed using two mobile phase: A, water containing 1% acetic acid; and B, 2-propanol. The gradient solvent system was as follows: 0–5 min, 0% B; 5.1–8.0 min, 100% B; 8.1–10.0 min, 0% B. The extraction from barley was repeated three times, and all data are expressed as the mean ± standard deviation (SD).

## Results and Discussion

### Isolation of 24MCA-FA from rice bran

Since OZ was first isolated from rice bran oil in 1954^11^, its presence has also been confirmed in various grains (e.g., wheat, barley and corn). There are about ten kinds of molecular species in OZ, and they are divided into TTA-OZ and PS-OZ^16^, but TTA-OZ is considered to be characteristic of rice. However, most recently, there are some reports suggesting the presence of 24MCA-FA, which belongs to TTA-OZ, in barley^12,13^, but the complete identification and quantification of 24MCA-FA in various cultivars of barley have not been carried out. In this study, to accurately evaluate the presence of 24MCA-FA in barley, we carried out the detailed structural analysis. The measurement of 24MCA-FA in various cultivars of barley was also conducted.

**Figure 2:**
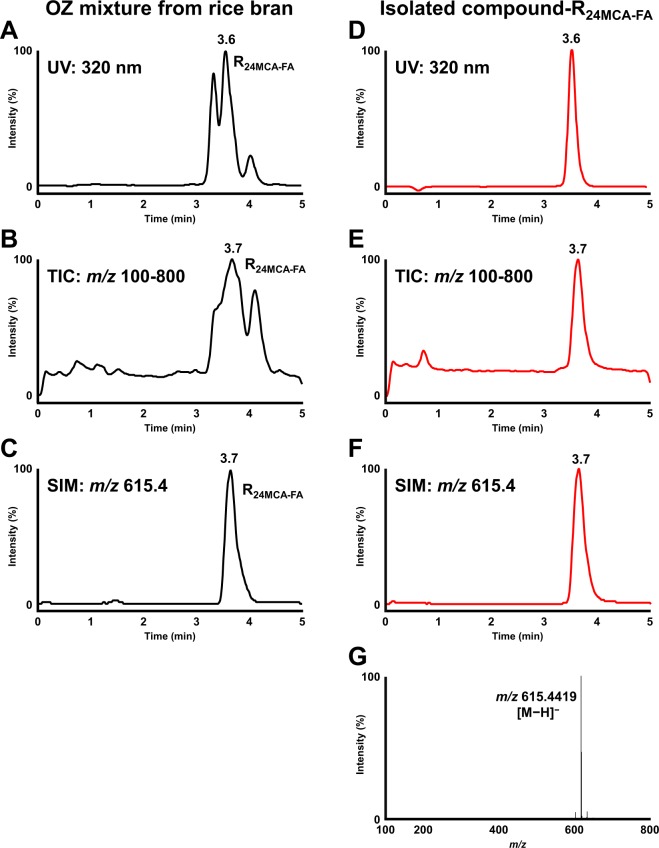
UV (320 nm) chromatogram, TIC (*m/z* 100-800), and SIM (*m/z* 615.4) obtained by injecting OZ mixture from rice bran (**A**–**C**) and the compound-R_24MCA-FA_ isolated from OZ mixture (**D**,**E**) into the HPLC-UV-MS. Mass spectra of the compound-R_24MCA-FA_ (**F**).

**Figure 3:**
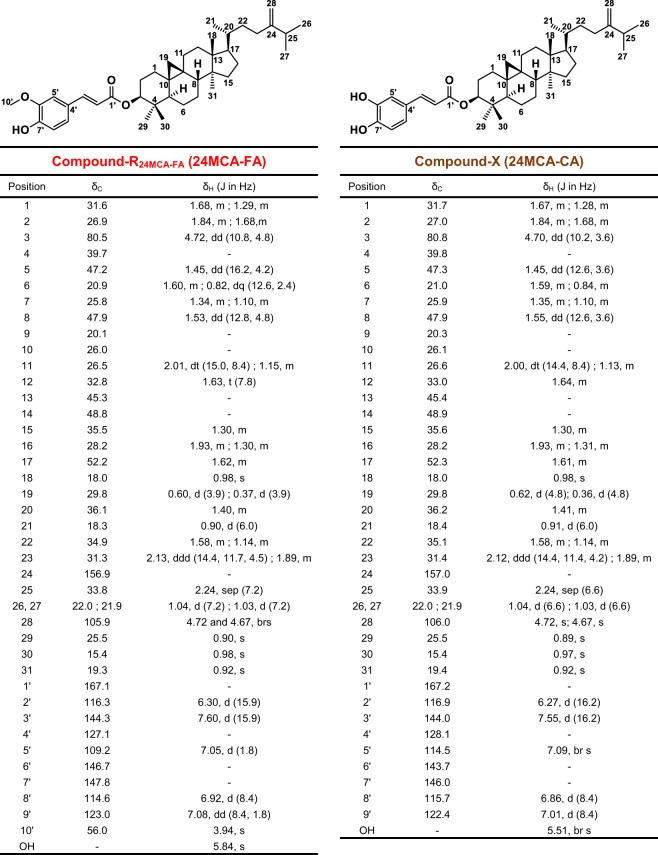
The full assignments of all proton and carbon of the isolated compound-R_24MCA-FA_ (24MCA-FA) and compound-X (24MCA-CA).

To determine the detailed structure of 24MCA-FA in barley, a standard of 24MCA-FA was needed, but as far as we know, there is no highly pure commercial standard of it. Therefore, in order to obtain the standard, first, we decided to isolate 24MCA-FA from OZ mixture derived from rice bran. A target compound considered as 24MCA-FA (Compound-R_24MCA-FA_) was confirmed by HPLC-UV-MS (Fig. 2A–C), then we tried to isolate the compound-R_24MCA-FA_. The compound-R_24MCA-FA_ (110 mg) was isolated as a white solid from rice OZ mixture. The isolated compound-R_24MCA-FA_ was subjected to a HPLC-UV-MS, HR-ESI-MS, HR-FAB-MS, NMR, and X-ray crystal structural analysis. The compound-R_24MCA-FA_ was detected as a single peak at 3.6 min in UV (320 nm) chromatogram, total ion current chromatogram (TIC) (*m/z* 100-800), and single ion chromatogram (SIM) (*m/z* 615.4) (Fig. 2D–F). Its molecular formula was determined to be C_41_H_60_O_4_ by HR-ESI-MS with *m*/z 615.4419 [M−H]^−^ (calcd. for C_41_H_59_O_4_ 615.4419) (Fig. 2G). And the fragmentation pattern of MS/MS analysis was shown in Supplementary Information 3. To investigate the sequence of the functional group, the compound R_24MCA-FA_ was subjected to HR-FAB-MS analysis. The presence of an ion at *m/z* 177.0553 ([C_10_H_9_O_3_]^+^) and *m/z* 439.3942 ([C_31_H_51_O]^+^) suggested that the compound-R_24MCA-FA_ contained feruloyl and 24-methylencycloartanol (24MCA) moieties. The ^13^C NMR spectra showed 41 carbon resonances, of which ten were assigned to the feruloyl moiety (Fig. 3). Inspection of various 1D and 2D NMR spectra (^1^H, ^13^C, COSY, HSQC, HMBC, NOESY) indicated the presence of carbonyl group at δ_C_ 167.1, two olefinic carbons at δ_C_ 116.3, and 144.3, six aromatic carbons at δ_C_ 109.2, 114.6, 123.1, 127.1, 146.7, and 147.8, and one oxymethyl carbon at δ_C_ 56.0, which supported the presence of feruloyl ester on the compound-R_24MCA-FA_ (Fig. 3) (Supplementary Information 3). In addition, the assumption that compound-R_24MCA-FA_ consisted of 24MCA was strengthened after comparing its NMR spectra with 24MCA literature data^17,18^, although the ^1^H and ^13^C NMR of complete structure of 24MCA-FA have not been reported in any literature yet. However, the comprehensive study about the structure determination of 24MCA-FA using NMR analysis is still not fully explored yet (only one study has reported isolation of 24MCA^19^), so it is crucial to perform full assignments of all proton and carbons of the compound-R_24MCA-FA_ to clarify whether its structure is 24MCA-FA. Then, the additional inspection of NMR data of the compound-R_24MCA-FA_ (Fig. 3) (Supplementary Information 3) enabled full assignments of the all proton and carbon of the compound-R_24MCA-FA_, which rationalized the structure of the compound-R_24MCA-FA_ to be 24MCA-FA. Moreover, the X-ray crystallography data of the compound-R_24MCA-FA_ (recrystallized from a mixed solvent system of hexane and chloroform) proved the structure of the compound-R_24MCA-FA_ to be 24MCA-FA (Supplementary Information 4). [additional spectroscopic data of 24MCA-FA: [α]^20^_D_ + 37.7 (*c* 0.33, CHCl_3_); IR: ν_max_ 3544 (w), 3413 (br), 1704 (s), 1514 (s), 1268 (s)] Thus, we decided to use it as a high-purity standard of 24MCA-FA (rice bran 24MCA-FA) in order to completely determine the structure of 24MCA-FA in barley. As for your reference, to the best of our knowledge, this study reported for the first time the detailed identification of 24MCA-FA by the combination of HPLC-UV-MS, HR-ESI-MS, HR-FAB-MS, NMR, and X-ray crystal structural analysis.

### Determination of the chemical structure of the compound-B_24MCA-FA_ and the compound-X from barley bran

**Figure 4:**
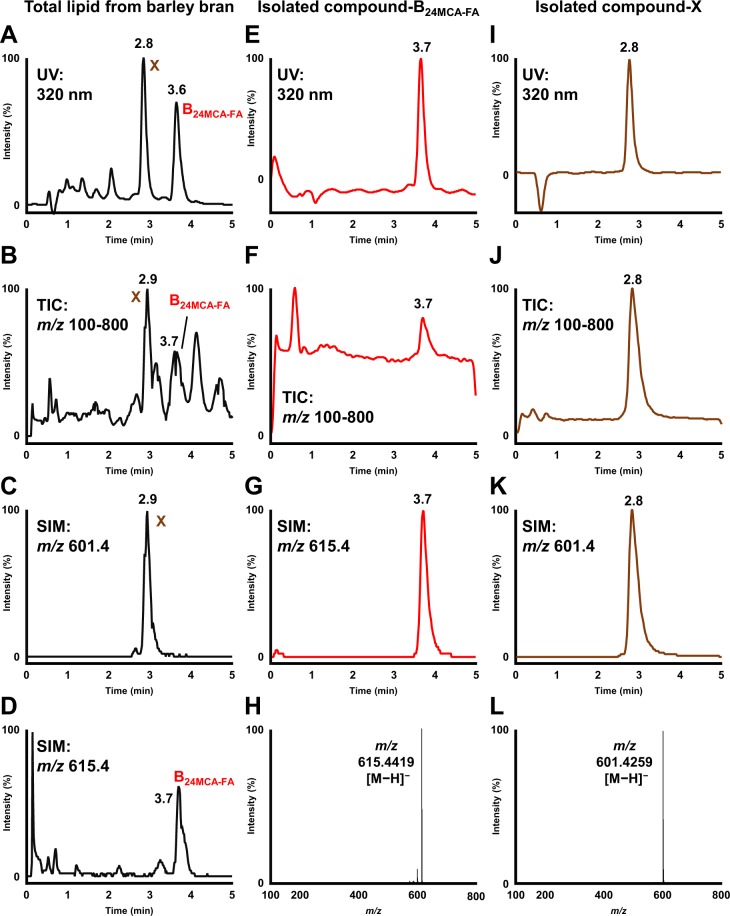
UV (320 nm) chromatogram, TIC (*m/z* 100-800), and SIM (*m/z* 615.4 or 601.4) obtained by injecting total lipid from barley bran (**A**–**D**), the compound-B_24MCA-FA_ isolated from barley bran (**E**–**G**), and the compound-X isolated from barley bran (**I**–**K**) into the HPLC-UV-MS. Mass spectra of the compound-B_24MCA-FA_ (**H**) and the compound-X (**L**).

Using the rice bran 24MCA-FA as a reference, both the standard and the total lipid fraction (hexane-extract) from barley bran sample were subjected to HPLC-UV-MS. Two large peaks were detected at 2.8 min (Peak 1) and 3.6 min (Peak 2) in the UV (320 nm) chromatogram of the barley bran sample (Fig. 4A). In addition, in mass spectra corresponding to the Peak 1 from the TIC, *m/z* 601.5 was mainly detected, and the Peak 2 showed *m/z* 615.4 (data not shown). Moreover, the SIM for *m/z* 601.5 and *m/z* 615.4 were coincided with the Peak 1 and the Peak 2, respectively (Fig. 4B–D). These results suggested the possibility of the presence of compound considered as 24MCA-FA (compound-B_24MCA-FA_) and another OZ molecular species (compound-X) in barley. To investigate the possibility, we tried to isolate these two compounds from barley bran using liquid-liquid extraction, flash-chromatography, and HPLC technique. From the isolation process, 0.77 mg of the compound-B_24MCA-FA_ was also isolated from 100 g of barley bran and detected as a single peak at 3.7 min in UV (320 nm) chromatogram, TIC (*m/z* 100-800), and SIM (*m/z* 615.4) (Fig. 4E–G). We also isolated 3.7 mg of the compound-X from 300 g of barley bran. The compound-X was detected as a single peak at 2.8 min in UV (320 nm) chromatogram, TIC (*m/z* 100-800), and SIM (*m/z* 601.4) (Fig. 4I–K). Then, each chemical structure was determined as described below.

Since the compound-B_24MCA-FA_ (*m/z* 615.4) corresponded to the rice bran 24MCA-FA (*m/z* 615.4), we tried to confirm whether the compound-B_24MCA-FA_ was 24MCA-FA by determining the chemical structure of the compound-B_24MCA-FA_. From HR-ESI-MS analysis, the compound-B_24MCA-FA_ was observed to have *m/z* 615.4419 [M−H]^−^ and it was consistent with the result from rice bran 24MCA-FA (*m/z* 615.4419 [M−H]^−^) (Figs 2G and 4H). The result of ^1^H NMR analysis of compound-B_24MCA-FA_ was identical to the standard 24MCA-FA. From these results, the existence of 24MCA-FA in barley was confirmed by chemical structure analysis for the first time.

Subsequently, we tried to determine the chemical structure of the compound-X. Our hypothesis was the compound-X might represent to CA-FA (i.e., ferulic acid esters of cycloartenol (CA)), a typical OZ molecular species of rice, since both of them have the same molecular ion mass (*m/z* 601.5). However, the HPLC result showed the different retention time between the compound-X (2.8 min) and CA-FA (3.4 min) (data not shown), which is assumed that the compound-X was not CA-FA, thus we carried out further analysis for chemical structure. The compound-X from barley bran was obtained as white solid. Its molecular formula was determined to be C_40_H_58_O_4_ by negative-ion HR-ESI-MS with *m/z* 601.4259 [M−H]^−^ (calcd. for C_40_H_57_O_4_ 601.4262) (Fig. 4L). And the fragmentation pattern of MS/MS analysis was shown in Supplementary Information 5. To investigate the sequence of the functional group, the compound-X was subjected to HR-FAB-MS analysis. The presence of an ion at *m/z* 163.0398 ([C_9_H_7_O_3_]^+^) and *m/z* 439.3944 ([C_31_H_51_O]^+^) suggested that the compound-X contained caffeoyl and 24MCA moieties. ^1^H and ^13^C NMR of the compound-X was very similar to 24MCA-FA except for the absence of oxymethyl group. Ultimately, inspection of various 1D and 2D NMR spectra (^1^H, ^13^C, COSY, HSQC, HMBC, NOESY) of the compound-X (Fig. 3) (Supplementary Information 5), rationalized the structure of the compound-X to be a caffeic acid esters of 24-methylenecycloartanol (24MCA-CA). [additional spectroscopic data of 24MCA-CA: [α]^16^_D_ + 35.9 (*c* 0.12, CHCl_3_); IR: ν_max_ 3479 (w), 3292 (br), 1685 (s), 1442 (m), 1278 (vs)] Even a lot of studies have been reported about the presence and function of caffeic acid and its esters in several plants, but to the best of our knowledge, only one study focused on the presence of caffeic acid ester of triterpene alcohols, especially for 24-methylenecycloartanyl (i.e., 24MCA-CA). The study conducted by Takagi *et al*. in 1980 has reported the presence of 24MCA-CA in canary seed by using TLC and GC/MS analysis^20^, however, the detailed chemical structural determination has not been examined in the study. Thus, our study is the first report that confirms the presence of 24MCA-CA in barley and determines its chemical structure by using HPLC-UV-MS, HR-ESI-MS, HR-FAB-MS, and NMR technics.

Through these findings, it is revealed that barley bran contains 24MCA-FA, which previously considered as a specific OZ species in rice. This study has also successfully proved the existence of 24MCA-CA in barley which has been rarely reported before, by verifying its chemical structure. As the presence of 24MCA-FA and 24MCA-CA in barley has been clearly clarified, the quantification in various cultivars of barley has also been conducted.

### Quantification of 24MCA-FA and 24MCA-CA in various cultivars of barley

Each barley cultivar has a characteristic type (e.g., hulled/hull-less and two-rowed/six-rowed)^21,22^. In this study, the whole and pearled barley grains of eight barley cultivars (‘Mikamo Golden’ (hulled two-rowed barley), ‘Nishinohoshi’ (hulled two-rowed barley), ‘Kashima Goal’ (hulled six-rowed barley), ‘Minorimugi’ (hulled six-rowed barley), ‘Kirarimochi’ (hull-less two-rowed barley), ‘Beau Fiber’ (hull-less two-rowed barley), ‘Ichibanboshi’ (hull-less six-rowed barley), and ‘Sanukihadaka’ (hull-less six-rowed barley)) (i.e., total sixteen samples) (Supplementary Information 1) were analyzed. In order to quantify 24MCA-FA and 24MCA-CA, the total lipid content was first extracted by liquid-liquid extraction method. In this study, we compared the total lipid content from the whole grain and the pearled grain. In the whole grain, the total lipid content was approximately 23.6–39.1 mg/g wet weight. Since the bran layer contains a lot of lipids, the total lipid content of the whole grain was 2 times higher than the one that was extracted from pearled grain (approximately 7.1–18.6 mg/g wet weight). These results were also comparable to the previous study^23^.

**Figure 5:**
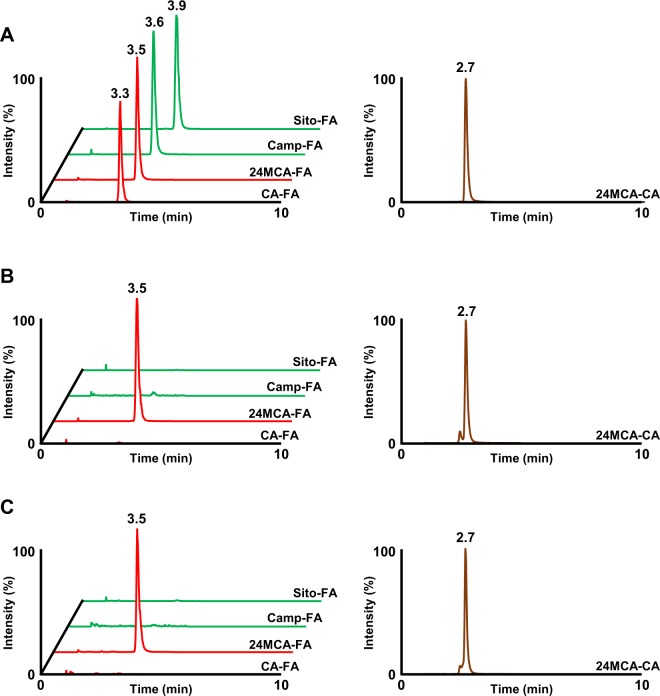
HPLC-MS/MS chromatograms of standard OZ mixture (containing CA-FA, 24MCA-FA, Camp-FA, and Sito-FA) and 24MCA-CA (**A**), whole grain of barley (e.g., ‘Mikamo Golden’) (**B**), and pearled grain of barley (e.g.,’Mikamo Golden’) (**C**).

**Figure 6:**
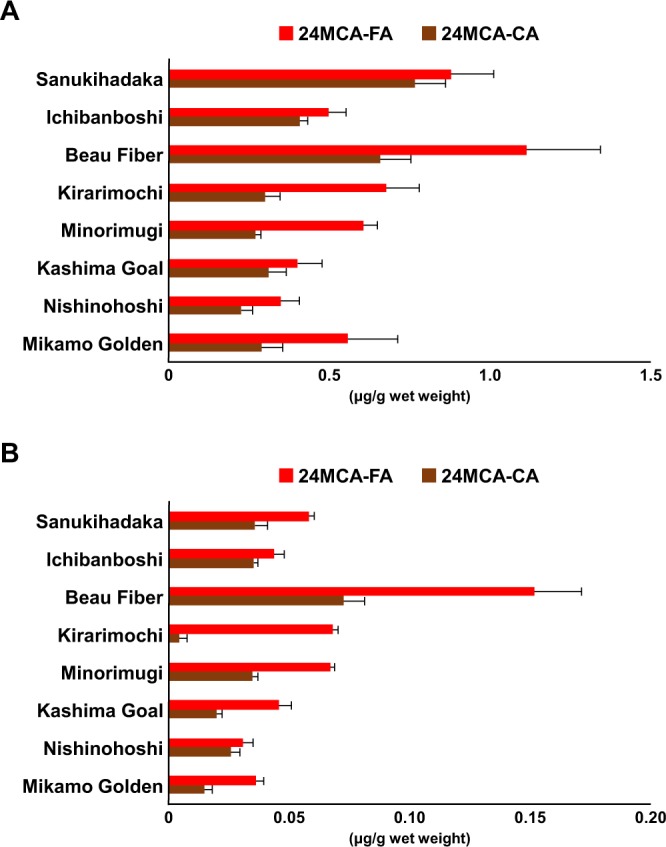
The concentration (μg/g wet weight) of 24MCA-FA and 24MCA-CA of the barley cultivars (i.e., ‘Mikamo Golden’, ‘Nishinohoshi’, ‘Kashima Goal’, ‘Minorimugi’, ‘Kirarimochi’, ‘Beau Fiber’, ‘Ichibanboshi’, and ‘Sanukihadaka’) of whole grain (**A**) and pearled grain (**B**). All data are expressed as mean ± SD (n = 3).

As the total lipid content has been successfully extracted, the OZ fraction then can be prepared by solid phase extraction. 24MCA-FA and 24MCA-CA were analyzed by HPLC-MS/MS method. As a result, 24MCA-FA and 24MCA-CA were found in every barley type of this study, concluded that these two species exist in barley over the cultivars (Figs 5 and 6). The concentration of 24MCA-FA in barley was 0.35–1.11 μg/g wet weight in whole grain (Fig. 6A) and 0.030–0.152 μg/g wet weight in pearled grain (Fig. 6B). The whole grain contained about 6.8–13.8 times higher than the 24MCA-FA of pearled grain, and it was found that 24MCA-FA was mostly fractionated into the bran. The concentration of 24MCA-FA in each barley cultivars were similar except for ‘Beau Fiber’. The pearled grain of ‘Beau Fiber’ has contained a relatively high concentration of 24MCA-FA. Maybe the reason is that grains of ‘Beau Fiber’ has a wrinkle-shaped particle, so it is considered that the part of bran tends to remain after pearled, and as a result, 24MCA-FA might have been high concentration. The presence of 24MCA-FA has not been reported for grains other than rice and barley^9^. In the case of rice, the content of 24MCA-FA in brown rice is reported to be 72.8–174.3 μg/g dry weight^24^ and in hull rice to be 9.7–20.4 μg/g wet weight^25^. Although the concentration of 24MCA-FA in barley is about 1/100-fold that of rice, barley is important as the only source of 24MCA-FA other than rice. The concentration of 24MCA-CA was 0.23–0.77 μg/g wet weight in whole grain and 0.004–0.072 μg/g wet weight in pearled grain (Fig. 6). 24MCA-CA was also abundantly contained in bran, and the whole grain contained 24MCA-CA about 1.5–13.3 times higher than pearled grain. From this result, it can be concluded that 24MCA-CA was less than 24MCA-FA. Remarkably, regardless of barley variety, common OZ molecular species (e.g., CA-FA, Camp-FA, and Sito-FA) other than 24MCA-FA were not detected (Fig. 5). This result suggested that OZ species in barley was limited to 24MCA-FA. Through this study, the existence of 24MCA-FA and 24MCA-CA in various barley cultivars was confirmed for the first time.

Regarding the biosynthesis, 24MCA-FA might be synthesized from CA-FA in barley, because 24MCA is known to be synthesized by cycloartenol-C24-methyltransferase from CA^26,27^. However, CA-FA has not been detected in barley cultivars which were evaluated in this study. 24MCA-FA might also be synthesized from 24MCA-CA, because caffeic acid is the precursor of ferulic acid in plants^28^. By further elucidating these possibilities, it may be helpful to have a better understanding about the biosynthetic pathway of OZ which has not been clarified yet.

## Conclusion

The presence of 24MCA-FA in barley and its chemical structure were determined by using HPLC-UV-MS, HR-MS, and NMR. We revealed that barley bran certainly contains 24MCA-FA, which previously considered as a specific OZ species in rice. This study also discovers that 24MCA-CA, which has rarely been reported before, exists in barley bran, and successfully determines its chemical structure. Furthermore, the quantification of 24MCA-FA and 24MCA-CA in various cultivars of barley was carried out, and the concentrations of 24MCA-FA and 24MCA-CA were clarified in all cultivars of barley analyzed in this study. The findings obtained in this study could be expected to bring new insight into the elucidation of the biosynthetic pathway of OZ. Moreover, this study opens the new possibility that TTA-OZ might exist in other plants as well as rice, thus we are going to explore this in the future study. Since the absorption, metabolism and physiological actions of OZ could be different in OZ molecular species^29–33^, it would be crucial to further understand the OZ molecular species distribution data in plants. Through these findings, it opens the possibility to use barley as a new source of 24MCA-FA and 24MCA-CA. Barley contains a lot of functional compounds (e.g., flavanols)^34^ and is used for not only staple food but also many food productions (e.g., bread and beer). More recently, although limited to the cell experiments, it has been reported that 24MCA-FA has anti-cancer effects on human breast and lung cancer cells^30,35^. Considering the increasing utilization of barley in food production, this study provides the beneficial information of 24MCA-FA and 24MCA-CA of barley, thus contributes to future application of barley as a source of 24MCA-FA and 24MCA-CA.

## Supplementary information

Supplementary Information

## References

[1] Kim SP, Kang MY, Nam SH, Friedman M (2012). Dietary rice bran component gamma-oryzanol inhibits tumor growth in tumor-bearing mice. Molecular nutrition & food research.

[2] Wilson TA, Nicolosi RJ, Woolfrey B, Kritchevsky D (2007). Rice bran oil and oryzanol reduce plasma lipid and lipoprotein cholesterol concentrations and aortic cholesterol ester accumulation to a greater extent than ferulic acid in hypercholesterolemic hamsters. The Journal of nutritional biochemistry.

[3] Juliano C, Cossu M, Alamanni MC, Piu L (2005). Antioxidant activity of gamma-oryzanol: mechanism of action and its effect on oxidative stability of pharmaceutical oils. International journal of pharmaceutics.

[4] Wang O (2015). Effects of ferulic acid and gamma-oryzanol on high-fat and high-fructose diet-induced metabolic syndrome in rats. PloS one.

[5] Ishihara M (1982). [Clinical effect of gamma-oryzanol on climacteric disturbance -on serum lipid peroxides (author’s transl)]. Nihon Sanka Fujinka Gakkai Zasshi.

[6] Minatel Igor, Francisqueti Fabiane, Corrêa Camila, Lima Giuseppina (2016). Antioxidant Activity of γ-Oryzanol: A Complex Network of Interactions. International Journal of Molecular Sciences.

[7] Ghatak SB, Panchal SJ (2012). Investigation of the immunomodulatory potential of oryzanol isolated from crude rice bran oil in experimental animal models. Phytotherapy research: PTR.

[8] Ismail N (2014). Mechanistic basis for protection of differentiated SH-SY5Y cells by oryzanol-rich fraction against hydrogen peroxide-induced neurotoxicity. BMC complementary and alternative medicine.

[9] Jiang Y, Wang T (2005). Phytosterols in cereal by-products. Journal of the American Oil Chemists’ Society.

[10] Norton RA (1995). Quantitation of steryl ferulate and p-coumarate esters from corn and rice. Lipids.

[11] Kobayashi E (2016). Presence of orally administered rice bran oil gamma-oryzanol in its intact form in mouse plasma. Food & function.

[12] Lee YJ (2017). Changes in the Functional Components of Barley Produced from Different Cultivars and Germination Periods. Cereal Chemistry.

[13] Tsuzuki W (2018). The unique compositions of steryl ferulates in foxtail millet, barnyard millet and naked barley. Journal of Cereal Science.

[14] Kobayashi E (2019). Evaluation of γ-oryzanol Accumulation and Lipid Metabolism in the Body of Mice Following Long-Term Administration of γ-oryzanol. Nutrients.

[15] Kokumai T (2019). Comparison of Blood Profiles of γ-Oryzanol and Ferulic Acid in Rats after Oral Intake of γ-Oryzanol. Nutrients.

[16] Xu Z, Godber JS (1999). Purification and Identification of Components of γ-Oryzanol in Rice Bran Oil. Journal of agricultural and food chemistry.

[17] Guo DA, Venkatramesh M, Nes WD (1995). Developmental regulation of sterol biosynthesis in Zea mays. Lipids.

[18] Yasukawa K, Akihisa T, Kimura Y, Tamura T, Takido M (1998). Inhibitory effect of cycloartenol ferulate, a component of rice bran, on tumor promotion in two-stage carcinogenesis in mouse skin. Biological & pharmaceutical bulletin.

[19] Hirose Y, Imai Y, Kondo T (1989). Conformational Analysis of Cycloartenol, 24-Methylenecycloartanol and Their Derivatives AU - Yoshida, Kumi. Agricultural and Biological Chemistry.

[20] Takagi T, Iida T (1980). Antioxidant for fats and oils from canary seed: Sterol and triterpene alcohol esters of caffeic acid. Journal of the American Oil Chemists Society.

[21] Holtekjølen AK, Kinitz C, Knutsen SH (2006). Flavanol and Bound Phenolic Acid Contents in Different Barley Varieties. Journal of agricultural and food chemistry.

[22] Holtekjølen AK, Uhlen AK, Bråthen E, Sahlstrøm S, Knutsen SH (2006). Contents of starch and non-starch polysaccharides in barley varieties of different origin. Food Chemistry.

[23] Czuchajowska Z, Klamczynski A, Paszczynska B, Baik B-K (1998). Structure and Functionality of Barley Starches. Cereal Chemistry.

[24] Miller A, Engel KH (2006). Content of gamma-oryzanol and composition of steryl ferulates in brown rice (Oryza sativa L.) of European origin. Journal of agricultural and food chemistry.

[25] Kim HW (2013). Evaluation of Î³-oryzanol content and composition from the grains of pigmented rice-germplasms by LC-DAD-ESI/MS. BMC Research Notes.

[26] Itoh T, Tamura T, Matsumoto T (1974). Sterols, methylsterols, and triterpene alcohols in three Theaceae and some other vegetable oils. Lipids.

[27] Schaller H (2003). The role of sterols in plant growth and development. Progress in lipid research.

[28] Ebel J (1974). Coordinated changes in enzyme activities of phenylpropanoid metabolism during the growth of soybean cell suspension cultures. Biochimica et Biophysica Acta (BBA) - General Subjects.

[29] Xu Z, Hua N, Godber JS (2001). Antioxidant activity of tocopherols, tocotrienols, and gamma-oryzanol components from rice bran against cholesterol oxidation accelerated by 2,2′-azobis(2-methylpropionamidine) dihydrochloride. Journal of agricultural and food chemistry.

[30] Kim HW (2015). 24-Methylenecycloartanyl ferulate, a major compound of γ-oryzanol, promotes parvin-beta expression through an interaction with peroxisome proliferator-activated receptor-gamma 2 in human breast cancer cells. Biochemical and biophysical research communications.

[31] Miller A, Majauskaite L, Engel K-H (2004). Enzyme-catalyzed hydrolysis of γ-oryzanol. European Food Research and Technology.

[32] Nakayama S, Manabe A, Suzuki J, Sakamoto K, Inagaki T (1987). Comparative effects of two forms of gamma-oryzanol in different sterol compositions on hyperlipidemia induced by cholesterol diet in rats. Japanese journal of pharmacology.

[33] Islam MS (2009). Antioxidant, free radical-scavenging, and NF-kappaB-inhibitory activities of phytosteryl ferulates: structure-activity studies. Journal of pharmacological sciences.

[34] Kohyama N (2009). Effects of Phenolic Compounds on the Browning of Cooked Barley. Journal of agricultural and food chemistry.

[35] Doello S, Liang Z, Cho IK, Kim JB, Li QX (2018). Cytotoxic Effects of 24-Methylenecyloartanyl Ferulate on A549 Nonsmall Cell Lung Cancer Cells through MYBBP1A Up-Regulation and AKT and Aurora B Kinase Inhibition. Journal of agricultural and food chemistry.

