# Author Correction: Definitive evidence of the presence of 24-methylenecycloartanyl ferulate and 24-methylenecycloartanyl caffeate in barley

**DOI:** 10.1038/s41598-020-57690-8

**Published:** 2020-01-17

**Authors:** Junya Ito, Kazue Sawada, Yusuke Ogura, Fan Xinyi, Halida Rahmania, Tomoyo Mohri, Noriko Kohyama, Eunsang Kwon, Takahiro Eitsuka, Hiroyuki Hashimoto, Shigefumi Kuwahara, Teruo Miyazawa, Kiyotaka Nakagawa

**Affiliations:** 1 Food and Biodynamic Chemistry Laboratory, Graduate School of Agricultural Science, Tohoku University, Sendai, Miyagi 980-8572 Japan; 2 Tsuno Food Industrial CO., LTD., Ito-Gun, Wakayama 649-7194 Japan; 3 Laboratory of Applied Bioorganic Chemistry, Graduate School of Agricultural Science, Tohoku University, Sendai, Miyagi 980-8572 Japan; 4 Institute of Crop Science, National Agriculture and Food Research Organization, Tsukuba, Ibaraki 305-8518 Japan; 5 Research and Analytical Center for Giant Molecules, Graduate School of Science, Tohoku University, Sendai, Miyagi 980-8578 Japan; 6 Food and Health Science Research Unit, Graduate School of Agricultural Science, Tohoku University, Sendai, Miyagi 980-8572 Japan; 7 Food and Biotechnology Innovation Project, New Industry Creation Hatchery Center (NICHe), Tohoku University, Sendai, Miyagi 980-8579 Japan

Correction to: *Scientific Reports* 10.1038/s41598-019-48985-6, published online 29 August 2019

In the Supplementary Information file originally published with this Article, the dataset ‘Supplementary Information 4’ was omitted. This error has been corrected in the Supplementary Information file that now accompanies the Article.

