# Supplementary material for: Definitive evidence of the presence of 24-methylenecycloartanyl ferulate and 24-methylenecycloartanyl caffeate in barley
Source: Sci Rep. 2019 Aug 29;9:12572. doi: 10.1038/s41598-019-48985-6 (PMC6715696; doi:10.1038/s41598-019-48985-6)

Definitive evidence of the presence of 24-methylenecycloartanyl ferulate and 24-methylenecycloartanyl caffeate in barley

Junya Ito[1,#], Kazue Sawada[1,2,#], Yusuke Ogura[3], Fan Xinyi[1], Halida Rahmania[1], Tomoyo Mohri[3], Noriko Kohyama[4], Eunsang Kwon[5], Takahiro Eitsuka[1], Hiroyuki Hashimoto[2], Shigefumi Kuwahara[3], Teruo Miyazawa[6,7], Kiyotaka Nakagawa[1,*]

1. Food and Biodynamic Chemistry Laboratory, Graduate School of Agricultural Science, Tohoku University, Sendai, Miyagi, 980-8572, Japan
2. Tsuno Food Industrial CO., LTD., Ito-Gun, Wakayama, 649-7194, Japan
3. Laboratory of Applied Bioorganic Chemistry, Graduate School of Agricultural Science, Tohoku University, Sendai, Miyagi, 980-8572, Japan
4. Institute of Crop Science, National Agriculture and Food Research Organization, Tsukuba, Ibaraki, 305-8518, Japan
5. Research and Analytical Center for Giant Molecules, Graduate School of Science, Tohoku University, Sendai, Miyagi, 980-8578, Japan
6. Food and Health Science Research Unit, Graduate School of Agricultural Science, Tohoku University, Sendai, Miyagi, 980-8572, Japan
7. Food and Biotechnology Innovation Project, New Industry Creation Hatchery Center (NICHe), Tohoku University, Sendai, Miyagi, 980-8579, Japan

*Corresponding Author: nkgw@m.tohoku.ac.jp

Supplementary Information 1

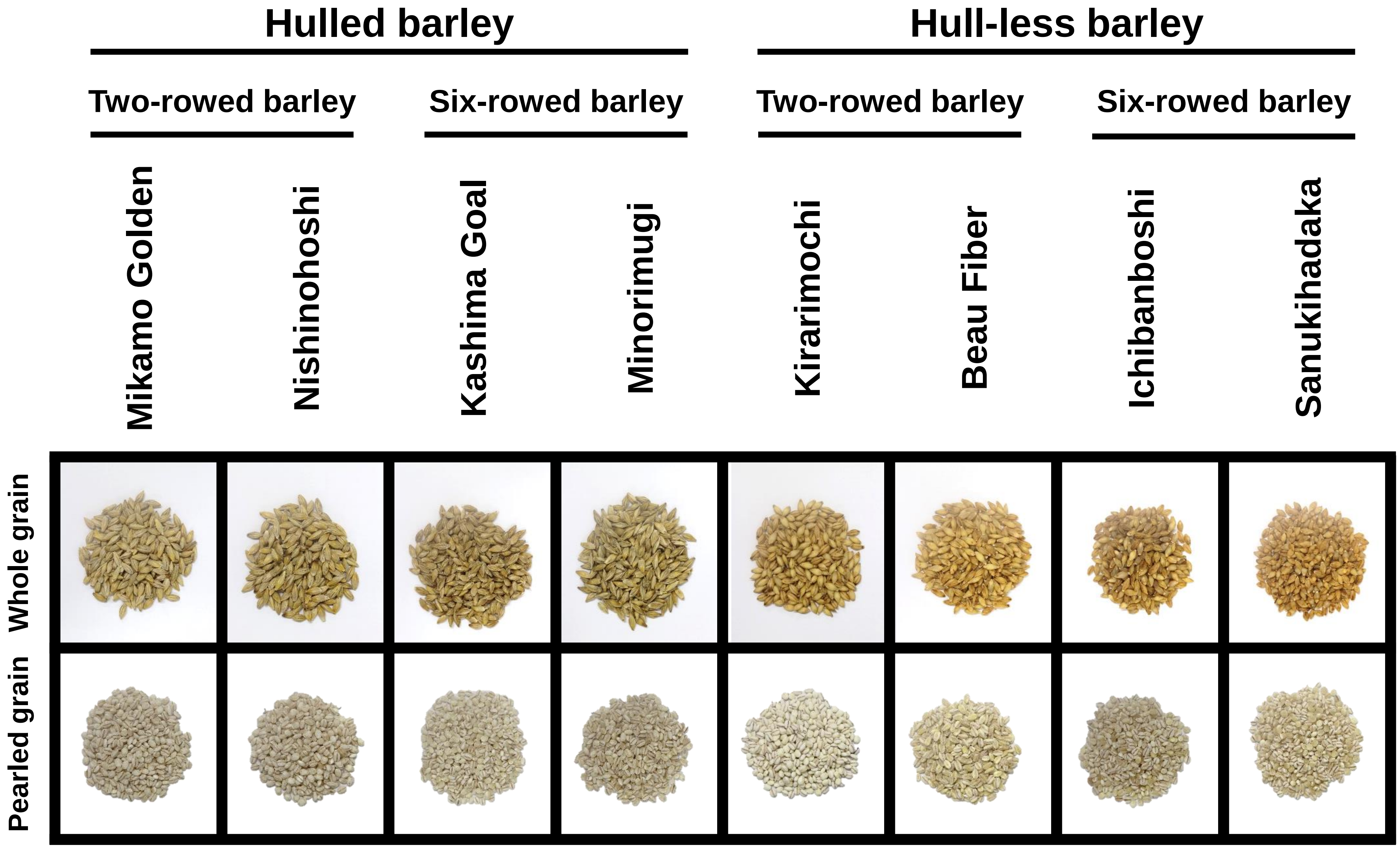

Supplementary Information 2

A. Analytical conditions and molecular ion mass of HR-ESI-MS analysis.

**Compound**	**24MCA-FA**	**24MCA-CA**
Theoretical molecular ion mass (*m/z*) $[M-H]^-$	615.4419	601.4262
Experimental molecular ion mass (*m/z*) $[M-H]^-$	615.4419	601.4259
Source	ESI	ESI
Ion polarity	Negative	Negative
Mass range (*m/z*)	100-800	100-800
End plate offset (v)	500	500
Capillary (v)	4000	4000
Nebulizer (Bar)	1.6	1.6
Dry gas (L/min)	8.0	8.0
Dry temp (^{o}C)	180	180
Funnel 1RF (Vpp)	400.0	400.0
Funnel 2RF (Vpp)	600.0	600.0
Hexapole RF (Vpp)	600.0	600.0
isCID energy (eV)	0.0	0.0
Ionenergy (eV)	20.0	20.0
Low mass (*m/z*)	70.00	70.00
Collision energy (eV)	10.0	10.0
Collision RF (Vpp)	1100.0	1100.0
Transfer time (μs)	55.0	55.0
Pre pulse storage (μs)	20.0	20.0

ESI: electrospray ionization.

Supplementary Information 2

B. Analytical conditions for HR-FAB-MS analysis.

Source	FAB
Ion polarity	Positive
Matrix	*m*-nitrobenzyl alcohol
FAB gas	Xenon
Acceleration voltage (kV)	8.0
FAB energy (keV)	6.0
Emission current (mA)	3.0

FAB: Fast atom bombardment.

Supplementary Information 2

C. Analytical conditions for HPLC-MS/MS analysis.

**Compound**	**CA-FA**	**24MCA-FA**	**Camp-FA**	**Sito-FA**	**24MCA-CA**
Precursor ion (*m/z*) $[M - H]^-$	601.4	615.4	575.4	589.4	601.4
Product ion (*m/z*)	586.3	600.3	560.3	574.3	160.8
Source	ESI	ESI	ESI	ESI	ESI
Ion polarity	Negative	Negative	Negative	Negative	Negative
Declustering potential (V)	-145.0	-145.0	-175.0	-155.0	155.0
Entrance potential (V)	-10.0	-10.0	-10.0	-10.0	-10.0
Collision energy (V)	-56.0	-58.0	-50.0	-52.0	-70.0
Collision cell exit potential (V)	-27.0	-29.0	-23.0	-31.0	-5.0
Curtain gas (psi)	20.0	20.0	20.0	20.0	20.0
Collision gas (psi)	5	5	5	5	5
Ion spray voltage (V)	-4500.0	-4500.0	-4500.0	-4500.0	-4500.0
Temperature (^oC)	700.0	700.0	700.0	700.0	700.0
Ion source gas 1 (psi)	50.0	50.0	50.0	50.0	50.0
Ion source gas 2 (psi)	80.0	80.0	80.0	80.0	80.0

ESI: electrospray ionization.

Supplementary Information 3

The fragmentation pattern of MS/MS analysis and the data of NMR analysis of compound-$R_{24MCA\text{-}FA}$.

A. **Fragmentation pattern of MS/MS analysis**
B. **^{1}H NMR (600 MHz, $CDCl_3$)**
C. **^{13}C NMR (150 MHz, $CDCl_3$)**
D. **COSY ($CDCl_3$)**
E. **HSQC ($CDCl_3$)**
F. **HMBC ($CDCl_3$)**
G. **NOESY ($CDCl_3$)**

A. Fragmentation pattern of MS/MS analysis

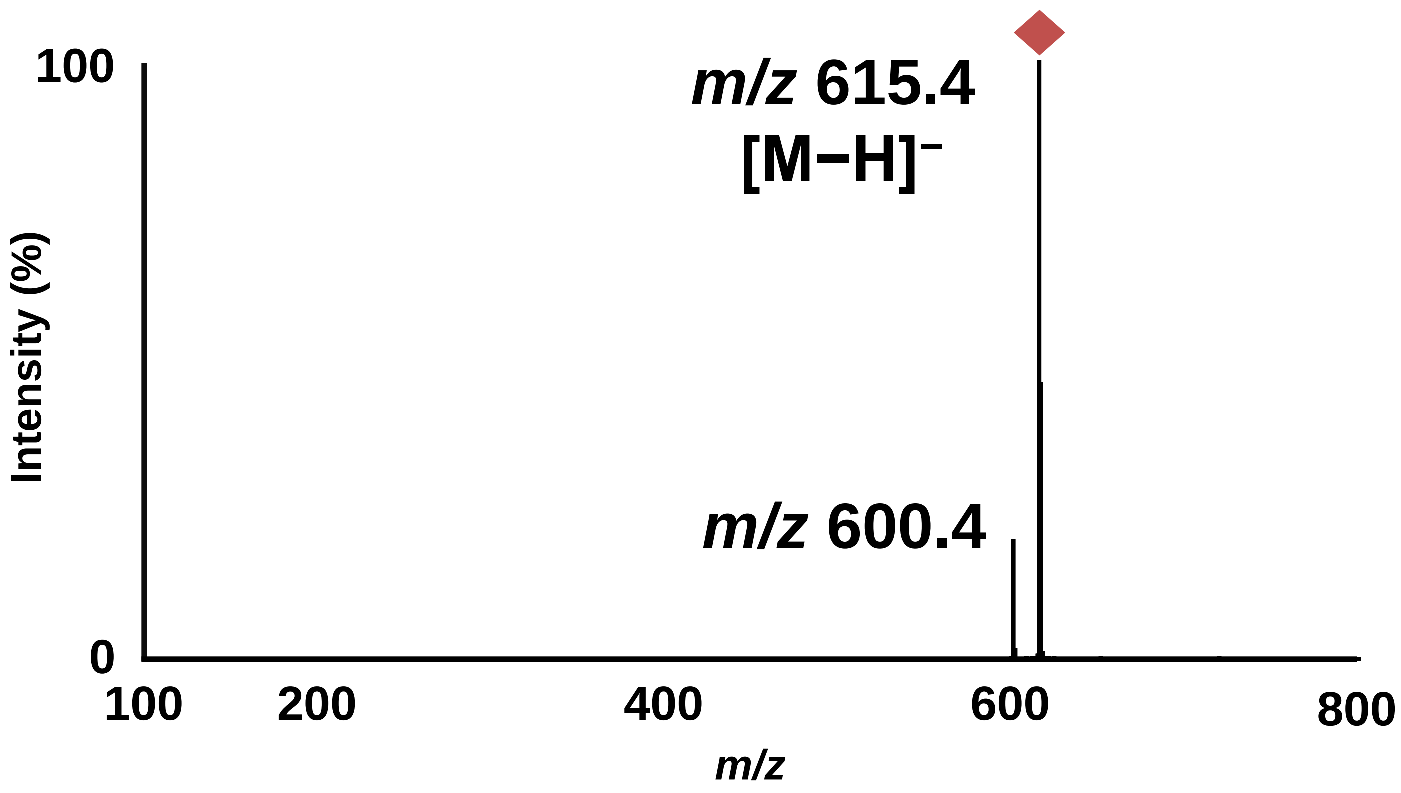

B. ^{1}H NMR (600 MHz, $CDCl_3$)

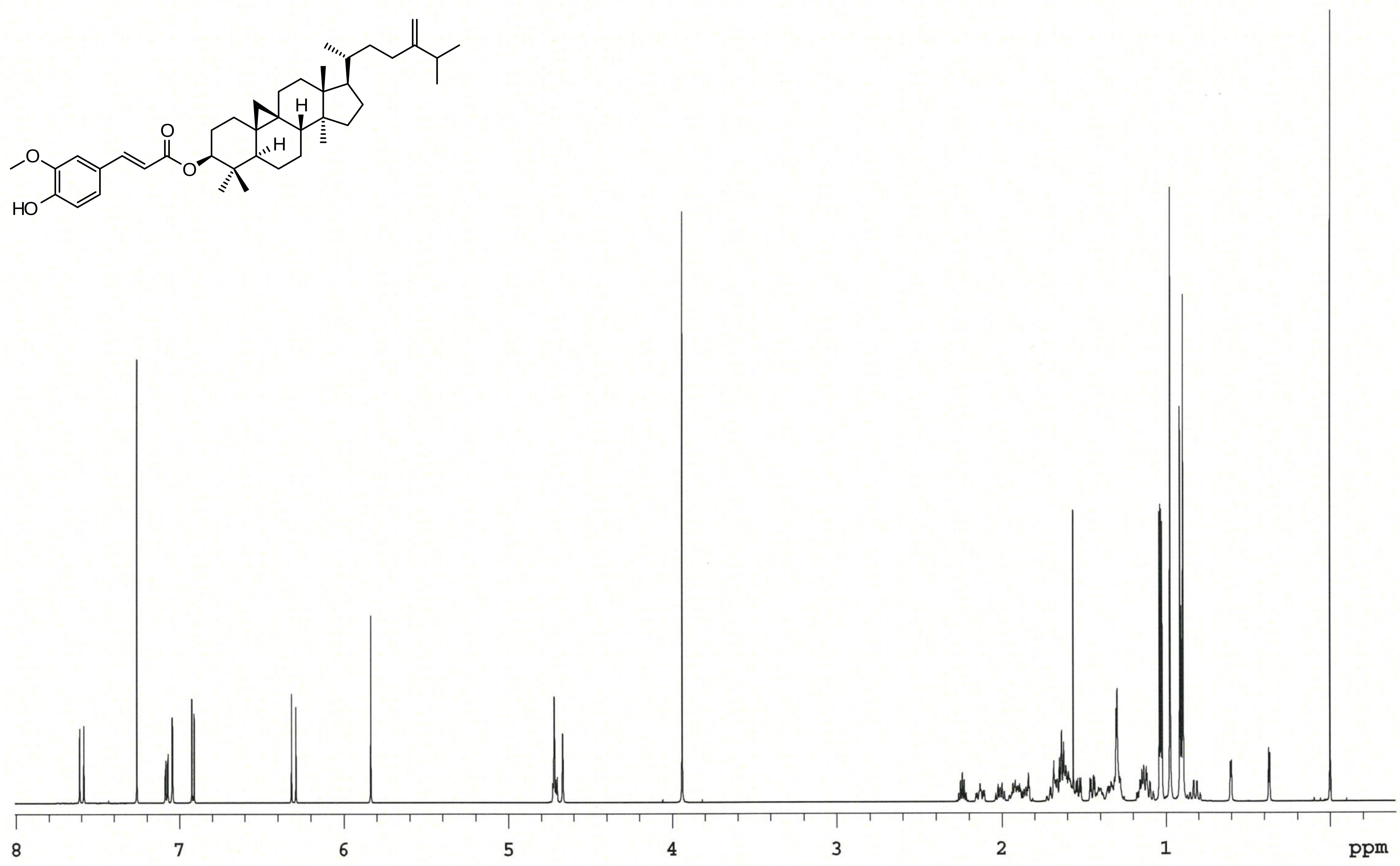

C. ^{13}C NMR (150 MHz, $CDCl_3$)

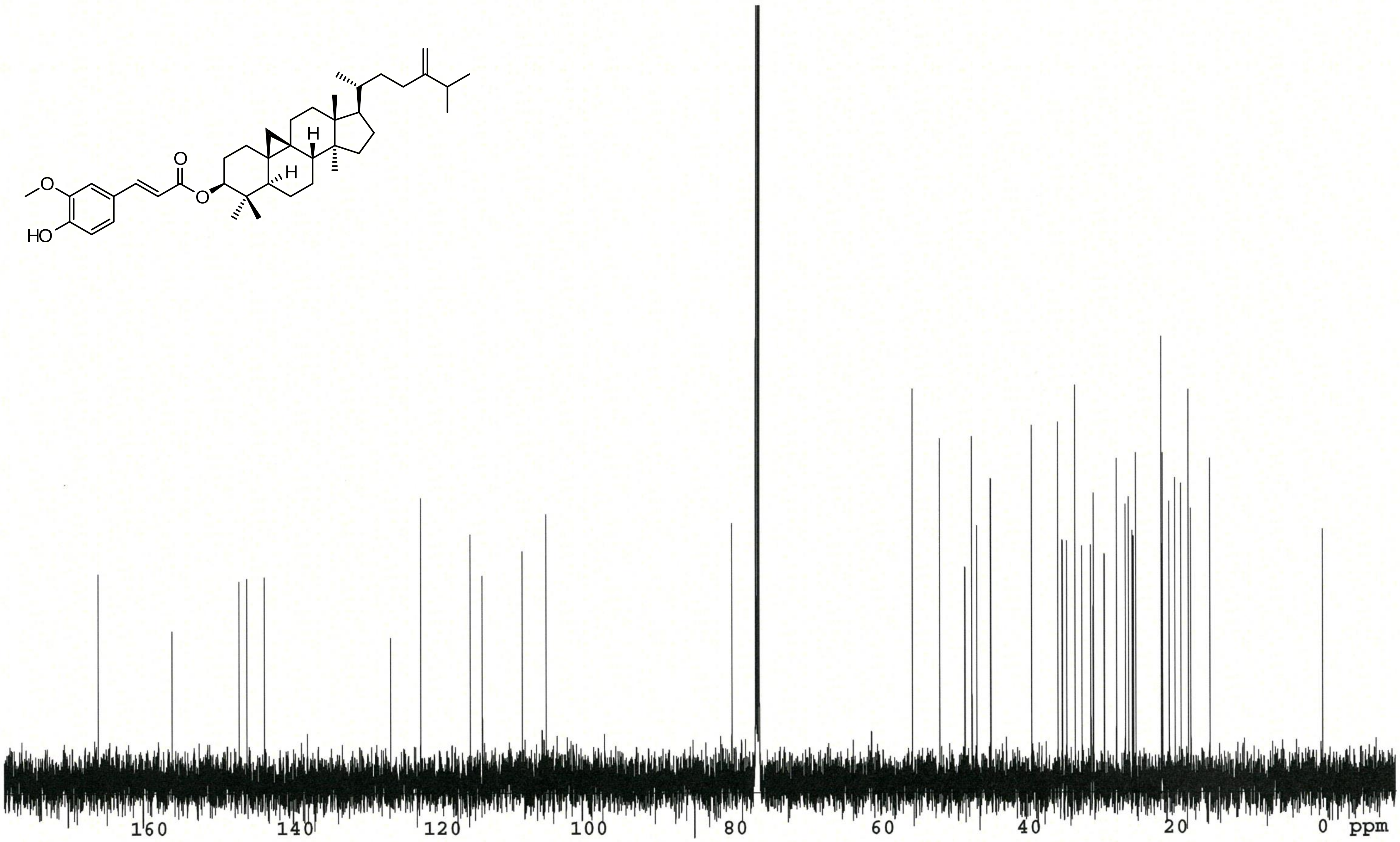

D. COSY ($CDCl_3$)

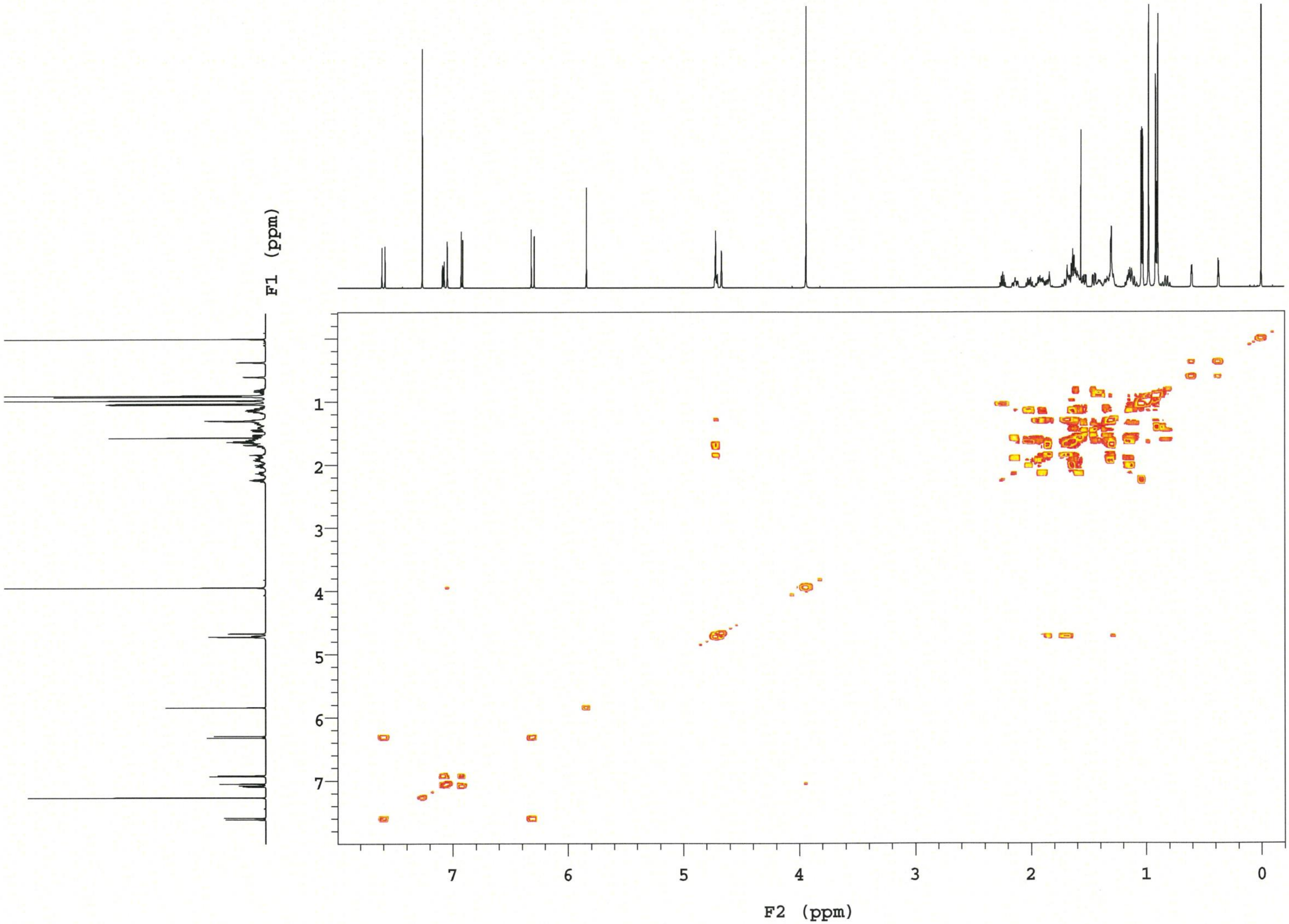

E. HSQC ($CDCl_3$)

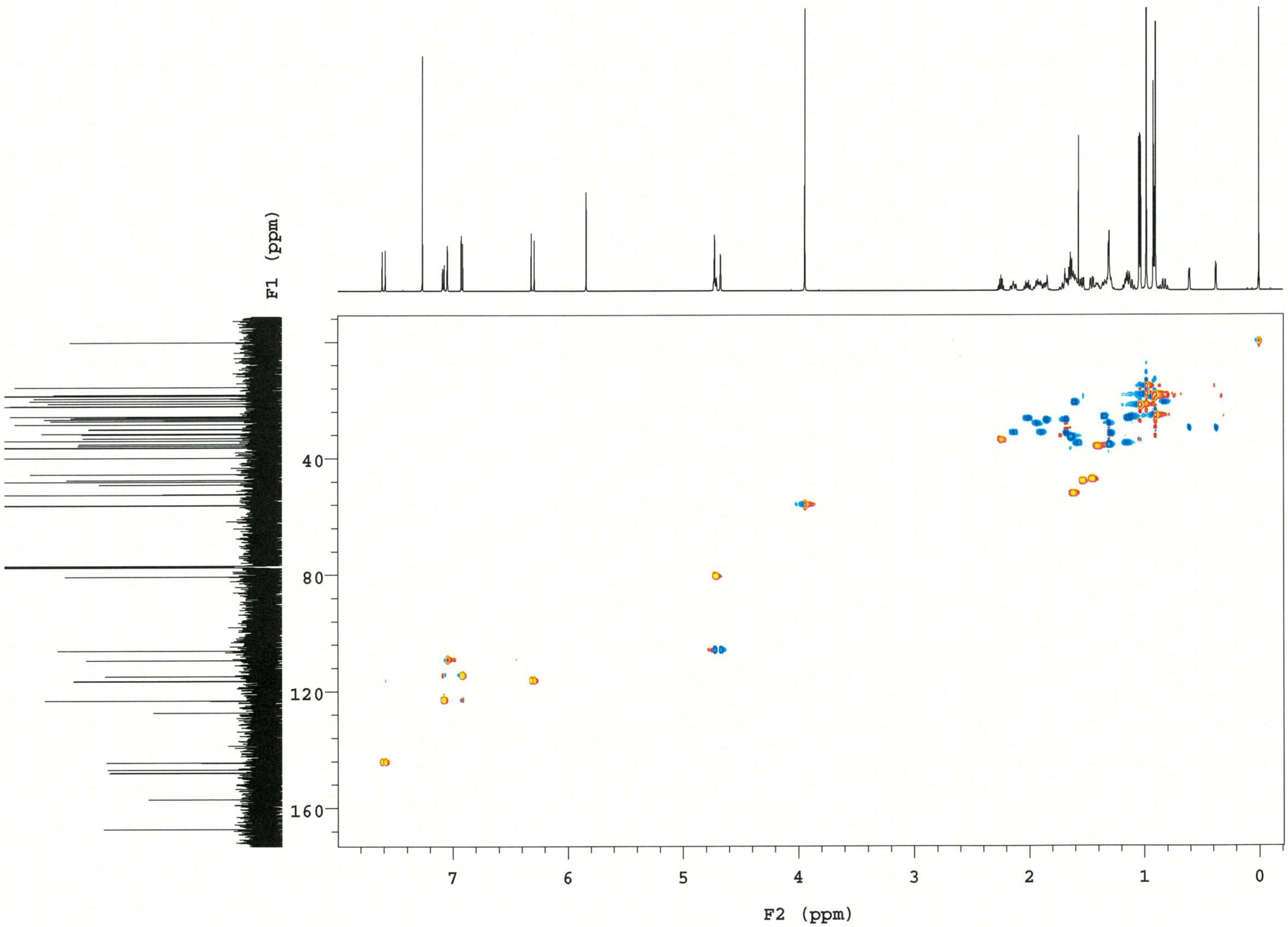

F. HMBC ($CDCl_3$)

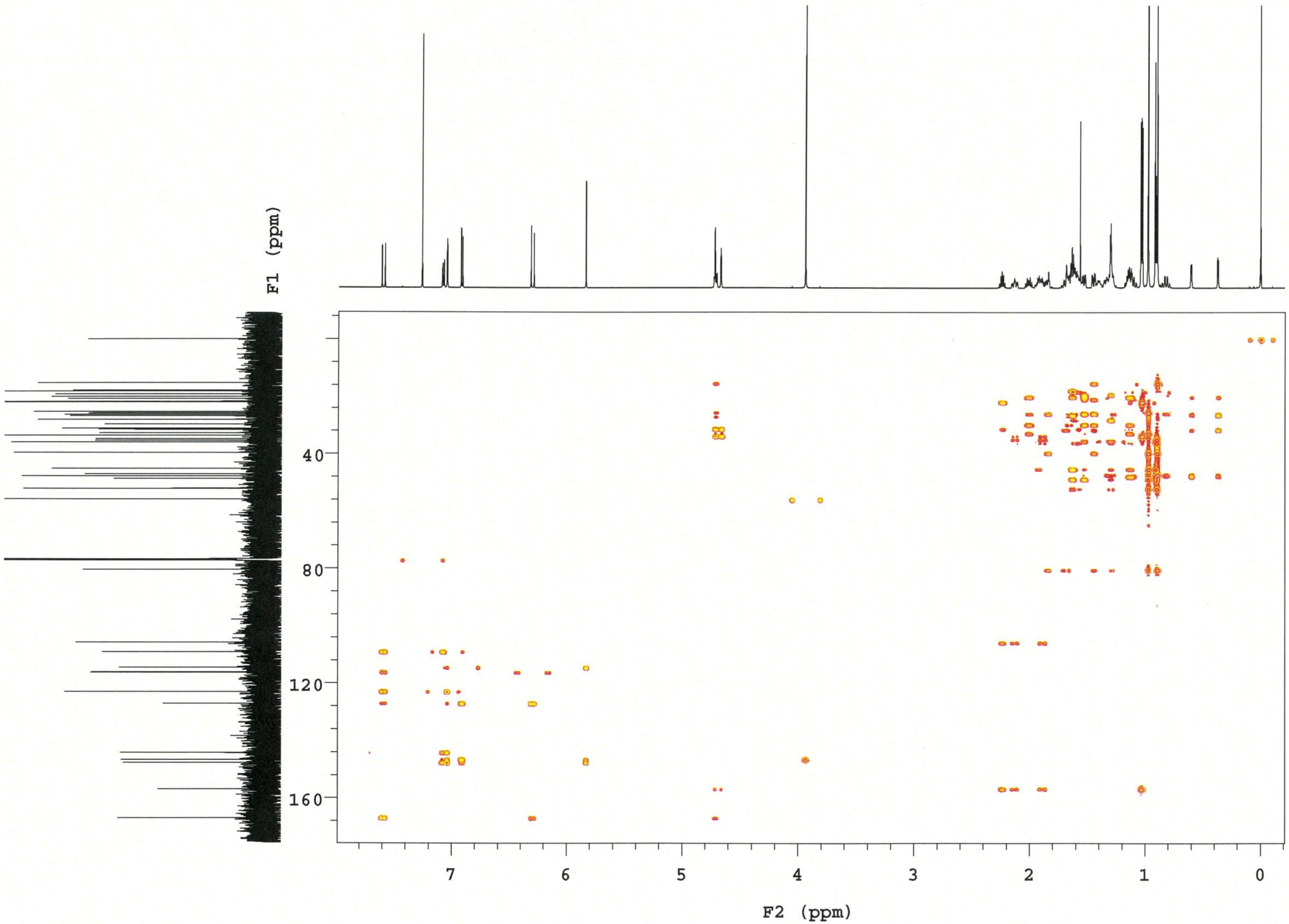

G. NOESY ($CDCl_3$)

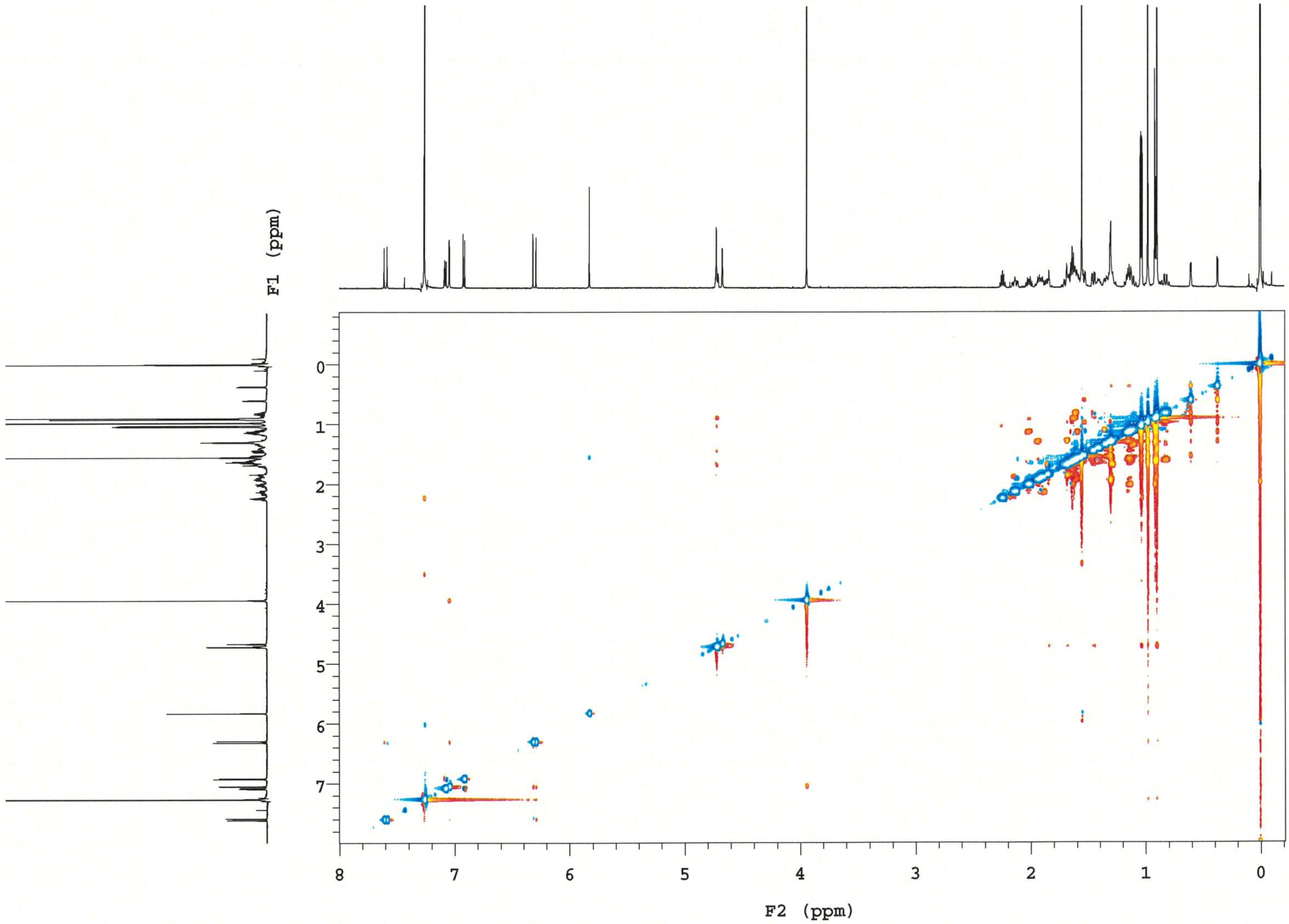

Supplementary Information 4

Experimental

The single crystal of 24MCA-FA suitable for the X-ray crystallography was grown by the slow solvent vapor diffusion method with hexane/chloroform (Figure S1).

The crystal was kept at 90.0 K during data collection. Using Olex2 [1], the structure was solved with the ShelXT [2] structure solution program using Intrinsic Phasing and refined with the ShelXL [3] refinement package using Least Squares minimisation. Crystallographic coordinates and analysis summary in CIF format have been deposited with the Cambridge Crystallographic Databank centre (CCDC). Its reference number is CCDC 1903270.

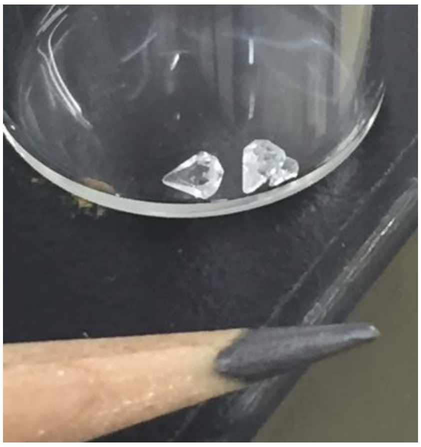

Figure S1. The crystal of 24MCA-FA.

1. Dolomanov, O.V., Bourhis, L.J., Gildea, R.J, Howard, J.A.K. & Puschmann, H. (2009), J. Appl. Cryst. 42, 339-341.
2. Sheldrick, G.M. (2015). Acta Cryst. A71, 3-8.
3. Sheldrick, G.M. (2015). Acta Cryst. C71, 3-8.

Crystal structure determination of 24MCA-FA

Crystal Data for $C_{41}H_{60}O_4$ (*M* =616.89 g/mol): tetragonal, space group $P4_32_12$ (no. 96), a = 10.690 Å, c = 64.332 Å, V = 7351.1 Å^3, Z = 8, T = 90.0 K, μ(CuKα) = 0.538 mm^{-1}, *Dcalc* = 1.115 g/cm^3, 78418 reflections measured (8.386° ≤ 2Θ ≤ 136.314°), 6718 unique (R_{int} = 0.1826, R_{sigma} = 0.0972) which were used in all calculations. The final R_1 was 0.0625 (I > 2σ(I)) and wR_2 was 0.1777 (all data).

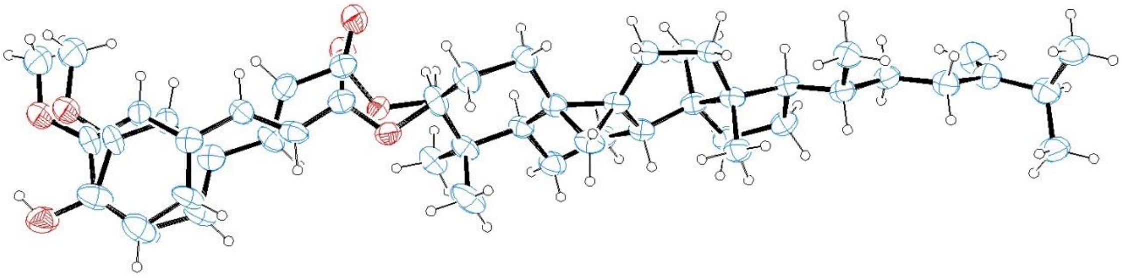

Figure S2. ORTEP drawing of 24MCA-FA showing thermal ellipsoids at the 30% probability level. In the crystal, the title compound has a disordered structure with two equally populated conformations of the cinnamic fragment.

24MCA-FA

Table S1. Crystal data and structure refinement for 24MCA-FA.

Empirical formula	$C_{41}H_{60}O_4$
Formula weight	616.89
Temperature/K	90.0
Crystal system	tetragonal
Space group	$P4_32_12$
a/Å	10.690
b/Å	10.690
c/Å	64.332
α/°	90
β/°	90
γ/°	90
Volume/Å^3	7351.1
Z	8
ρ_{calc}g/cm^3	1.115
μ/mm^{-1}	0.538
F(000)	2704.0
Crystal size/mm^3	0.15 × 0.15 × 0.01
Radiation	CuKα (λ = 1.54187)
2Θ range for data collection/°	8.386 to 136.314
Index ranges	-12 ≤ h ≤ 12, -12 ≤ k ≤ 12, -77 ≤ l ≤ 74
Reflections collected	78418
Independent reflections	6718 [R_{int} = 0.1826, R_{sigma} = 0.0972]
Data/restraints/parameters	6718/32/470
Goodness-of-fit on F^2	0.880
Final R indexes [I>=2σ (I)]	R_1 = 0.0625, wR_2 = 0.1515
Final R indexes [all data]	R_1 = 0.1077, wR_2 = 0.1777
Largest diff. peak/hole / e Å^{-3}	0.21/-0.14
Flack parameter	0.6(3)

Table S2. Fractional Atomic Coordinates (×10⁴) and Equivalent Isotropic Displacement Parameters (Å²×10³) for 24MCA-FA. U_{eq} is defined as 1/3 of of the trace of the orthogonalised U_{IJ} tensor.

Atom	*x*	*y*	*z*	U(eq)
O1A	-8705(9)	1185(11)	-4526(2)	61(3)
O1B	-8114(8)	1076(11)	-4504(2)	67(3)
O2A	-10796(9)	1396(9)	-4573.3(17)	89(3)
O2B	-10086(11)	1625(15)	-4618(3)	89(3)
O3A	-9390(15)	6937(14)	-5210(3)	92(3)
O3B	-9332(15)	7416(13)	-5270(3)	92(3)
O4	-6978(3)	7807(4)	-5217.4(5)	97.9(12)
C1	-8424(5)	-11169(5)	-2851.2(8)	94.4(17)
C1BA	-6195(5)	-11071(5)	-2973.1(8)	90.5(16)
C2	-7534(5)	-11426(5)	-3030.2(8)	77.8(15)
C4	-7996(5)	-10782(5)	-3225.5(8)	73.4(14)
C5	-8420(6)	-11491(6)	-3380.2(8)	100.2(18)
C6	-7954(5)	-9368(4)	-3232.7(7)	72.3(13)
C7	-8211(4)	-8785(4)	-3443.2(7)	68.1(13)
C8	-8027(4)	-7375(4)	-3461.7(7)	65.0(12)
C9	-8854(4)	-6669(4)	-3307.0(6)	71.5(13)
C10	-8248(4)	-6982(4)	-3690.5(6)	59.6(12)
C11	-7343(4)	-7676(5)	-3841.5(7)	70.3(13)
C12	-7117(4)	-6827(5)	-4030.2(7)	69.7(13)
C13	-8039(4)	-5724(4)	-4003.3(6)	62.3(12)
C14	-8123(4)	-5592(4)	-3761.4(7)	60.1(12)
C15	-6928(4)	-5024(5)	-3664.7(7)	70.8(13)
C16	-9233(4)	-4718(4)	-3709.0(7)	62.8(12)
C17	-9354(4)	-3588(4)	-3861.2(7)	61.9(12)
C18	-8274(4)	-3337(4)	-4011.2(7)	62.0(12)
C19	-7556(4)	-4496(4)	-4096.7(7)	64.2(12)
C20	-9293(4)	-6131(4)	-4099.2(6)	67.1(12)
C21	-7427(5)	-4499(5)	-4331.8(7)	73.3(14)
C22	-6944(5)	-3256(5)	-4411.1(7)	78.0(14)
C23	-7915(4)	-2237(4)	-4375.1(7)	60.6(12)
C24	-8369(4)	-2182(4)	-4151.0(7)	62.2(12)
C25	-7476(4)	-2198(4)	-3971.7(7)	72.0(13)
C26	-9497(5)	-1333(4)	-4131.0(7)	70.1(13)

C27	-9206(5)	-18(4)	-4217.0(7)	82.2(15)
C28	-7572(5)	-936(5)	-4466.3(7)	71.3(14)
C29	-7307(5)	-1030(5)	-4699.7(7)	85.2(16)
C30	-8741(5)	-95(4)	-4438.9(8)	78.3(15)
C39	-7377(6)	6759(7)	-5114.4(9)	91.3(18)
C41	-6434(6)	-363(6)	-4358.2(8)	108(2)
C61A	-9759(13)	1758(15)	-4594(3)	66(3)
C61B	-8949(12)	1838(14)	-4608(2)	66(3)
C62A	-9404(12)	2913(10)	-4718.0(16)	72(3)
C62B	-8370(20)	2890(20)	-4694(4)	71(6)
C63A	-8270(20)	3300(20)	-4751(4)	68(6)
C63B	-8950(13)	3761(11)	-4815.8(17)	64(3)
C64A	-7976(15)	4440(16)	-4883(3)	68(3)
C64B	-8424(15)	4860(17)	-4916(3)	68(3)
C65A	-8915(15)	5149(14)	-4983(2)	65(3)
C65B	-9147(17)	5601(14)	-5050(2)	65(3)
C66A	-8605(16)	6182(18)	-5101(4)	70(3)
C66B	-8648(16)	6636(18)	-5147(4)	70(3)
C67A	-6510(30)	5960(50)	-5013(10)	95(8)
C67B	-6580(30)	6160(50)	-4985(10)	95(8)
C68A	-6730(18)	4860(30)	-4894(6)	81(6)
C68B	-7156(17)	5160(30)	-4886(6)	81(6)
C69A	-10668(15)	6672(16)	-5193(3)	129(5)
C69B	-10568(15)	7211(16)	-5305(3)	129(5)

Table S3. Anisotropic Displacement Parameters (Å^2×10^3) for 24MCA-FA. The Anisotropic displacement factor exponent takes the form: -2π^2[h^{2}a*^{2}U$_{11}$+2hka*b*U$_{12}$+…].

Atom	U_{11}	U_{22}	U_{33}	U_{23}	U_{13}	U_{12}
O1A	57(7)	58(6)	69(5)	6(4)	-6(6)	8(6)
O1B	52(6)	61(5)	89(6)	-3(4)	-3(6)	0(5)
O2A	90(8)	64(5)	113(6)	13(4)	-6(6)	-10(5)
O2B	90(8)	64(5)	113(6)	13(4)	-6(6)	-10(5)
O3A	75(4)	70(10)	131(10)	14(6)	-18(5)	-21(6)
O3B	75(4)	70(10)	131(10)	14(6)	-18(5)	-21(6)
O4	68(2)	143(4)	83(2)	23(2)	9.6(19)	1(2)
C1	89(4)	99(4)	95(4)	-7(3)	4(3)	9(3)
C1BA	62(4)	84(4)	126(4)	5(3)	-6(3)	10(3)
C2	75(4)	67(3)	91(4)	-5(3)	10(3)	5(3)
C4	69(4)	64(3)	88(4)	-7(3)	4(3)	-1(3)
C5	126(5)	78(4)	96(4)	-14(3)	-1(4)	-22(4)
C6	70(3)	60(3)	87(3)	-3(3)	6(3)	0(3)
C7	56(3)	64(3)	84(3)	-3(3)	-5(3)	4(2)
C8	53(3)	64(3)	78(3)	-2(2)	-3(2)	0(2)
C9	71(3)	71(3)	72(3)	-8(3)	8(3)	5(3)
C10	49(3)	63(3)	67(3)	-6(2)	-4(2)	0(2)
C11	66(3)	68(3)	77(3)	-9(3)	-5(3)	10(2)
C12	60(3)	71(3)	78(3)	-11(3)	2(2)	13(3)
C13	54(3)	64(3)	69(3)	-13(2)	3(2)	7(2)
C14	48(3)	63(3)	70(3)	-6(2)	1(2)	-4(2)
C15	57(3)	71(3)	84(3)	-7(3)	-7(2)	-7(3)
C16	49(3)	62(3)	78(3)	-9(2)	9(2)	-4(2)
C17	59(3)	54(3)	73(3)	-9(2)	3(2)	1(2)
C18	48(3)	62(3)	76(3)	-11(2)	6(2)	-3(2)
C19	53(3)	71(3)	68(3)	-6(2)	7(2)	0(2)
C20	57(3)	71(3)	73(3)	-12(2)	-7(2)	6(2)
C21	62(3)	81(4)	77(3)	-5(3)	13(3)	15(3)
C22	66(3)	90(4)	78(3)	0(3)	20(3)	1(3)
C23	54(3)	56(3)	72(3)	-7(2)	6(2)	-6(2)
C24	58(3)	54(3)	74(3)	-10(2)	2(2)	-6(2)
C25	58(3)	74(3)	84(3)	-12(3)	7(3)	-12(3)
C26	76(4)	56(3)	78(3)	-2(2)	9(3)	-2(3)

C27	104(4)	55(3)	88(4)	-9(3)	9(3)	-11(3)
C28	69(3)	67(4)	78(3)	-8(3)	4(3)	-24(3)
C29	93(4)	81(4)	82(3)	1(3)	12(3)	-20(3)
C30	102(4)	51(3)	82(3)	1(3)	13(3)	-15(3)
C39	73(4)	131(6)	70(3)	-16(4)	15(3)	-24(4)
C41	107(5)	120(5)	96(4)	-5(3)	4(3)	-62(4)
C61A	61(8)	67(6)	70(5)	-12(4)	1(8)	7(9)
C61B	61(8)	67(6)	70(5)	-12(4)	1(8)	7(9)
C62A	73(8)	61(8)	81(7)	2(6)	-8(6)	12(6)
C62B	54(10)	59(15)	100(17)	-20(10)	13(9)	-12(8)
C63A	78(11)	52(15)	76(13)	-11(10)	16(9)	-19(10)
C63B	64(8)	58(8)	69(7)	-1(6)	10(6)	-4(7)
C64A	69(11)	65(11)	70(7)	-16(7)	5(7)	4(6)
C64B	69(11)	65(11)	70(7)	-16(7)	5(7)	4(6)
C65A	70(8)	58(10)	66(10)	8(5)	-6(6)	-4(6)
C65B	70(8)	58(10)	66(10)	8(5)	-6(6)	-4(6)
C66A	91(6)	47(13)	73(11)	11(7)	5(5)	-17(7)
C66B	91(6)	47(13)	73(11)	11(7)	5(5)	-17(7)
C67A	95(5)	124(13)	65(14)	2(13)	10(6)	-37(6)
C67B	95(5)	124(13)	65(14)	2(13)	10(6)	-37(6)
C68A	61(12)	108(14)	75(4)	1(8)	21(11)	-30(10)
C68B	61(12)	108(14)	75(4)	1(8)	21(11)	-30(10)
C69A	93(7)	91(12)	202(19)	38(7)	-29(10)	-15(7)
C69B	93(7)	91(12)	202(19)	38(7)	-29(10)	-15(7)

Table S4. Bond Lengths for 24MCA-FA.

Atom	Atom	Length/Å	Atom	Atom	Length/Å
O1A	C30	1.478(12)	C18	C24	1.530(6)
O1A	C61A	1.356(15)	C18	C25	1.508(6)
O1B	C30	1.481(12)	C19	C21	1.519(6)
O1B	C61B	1.381(13)	C21	C22	1.515(7)
O2A	C61A	1.182(14)	C22	C23	1.522(6)
O2B	C61B	1.238(14)	C23	C24	1.522(6)
O3A	C66A	1.361(15)	C23	C28	1.553(6)
O3A	C69A	1.399(17)	C24	C25	1.497(6)
O3B	C66B	1.363(15)	C24	C26	1.515(6)
O3B	C69B	1.359(16)	C26	C27	1.543(6)
O4	C39	1.370(7)	C27	C30	1.513(6)
C1	C2	1.519(6)	C28	C29	1.531(6)
C1BA	C2	1.525(7)	C28	C30	1.551(7)
C2	C4	1.515(6)	C28	C41	1.529(7)
C4	C5	1.331(6)	C39	C66A	1.453(16)
C4	C6	1.513(6)	C39	C66B	1.380(16)
C6	C7	1.516(6)	C39	C67A	1.42(2)
C7	C8	1.526(6)	C39	C67B	1.35(2)
C8	C9	1.530(6)	C61A	C62A	1.518(16)
C8	C10	1.548(6)	C61B	C62B	1.40(2)
C10	C11	1.559(5)	C62A	C63A	1.30(2)
C10	C14	1.560(6)	C62B	C63B	1.37(3)
C11	C12	1.535(6)	C63A	C64A	1.514(19)
C12	C13	1.547(6)	C63B	C64B	1.45(2)
C13	C14	1.565(6)	C64A	C65A	1.415(14)
C13	C19	1.533(6)	C64A	C68A	1.409(19)
C13	C20	1.538(6)	C64B	C65B	1.404(15)
C14	C15	1.545(6)	C64B	C68B	1.405(19)
C14	C16	1.547(6)	C65A	C66A	1.377(14)
C16	C17	1.560(6)	C65B	C66B	1.376(14)
C17	C18	1.528(6)	C67A	C68A	1.42(2)
C18	C19	1.558(6)	C67B	C68B	1.39(2)

Table S5. Bond Angles for 24MCA-FA.

Atom	Atom	Atom	Angle/°	Atom	Atom	Atom	Angle/°
C61A	O1A	C30	121.3(11)	C25	C24	C26	116.7(4)
C61B	O1B	C30	110.1(9)	C26	C24	C18	119.1(4)
C66A	O3A	C69A	116.3(12)	C26	C24	C23	110.9(4)
C69B	O3B	C66B	121.4(11)	C24	C25	C18	61.2(3)
C1	C2	C1BA	111.1(4)	C24	C26	C27	110.8(4)
C4	C2	C1	110.0(4)	C30	C27	C26	110.8(4)
C4	C2	C1BA	113.1(4)	C29	C28	C23	110.8(4)
C5	C4	C2	118.2(5)	C29	C28	C30	107.4(4)
C5	C4	C6	123.8(5)	C30	C28	C23	106.6(4)
C6	C4	C2	118.0(5)	C41	C28	C23	112.0(4)
C4	C6	C7	115.6(4)	C41	C28	C29	109.0(4)
C6	C7	C8	116.9(4)	C41	C28	C30	110.9(4)
C7	C8	C9	111.2(4)	O1A	C30	C27	108.3(6)
C7	C8	C10	108.8(4)	O1A	C30	C28	118.2(6)
C9	C8	C10	113.4(4)	O1B	C30	C27	111.8(7)
C8	C10	C11	111.6(3)	O1B	C30	C28	95.3(5)
C8	C10	C14	121.5(4)	C27	C30	C28	113.8(4)
C11	C10	C14	102.6(3)	O4	C39	C66A	131.1(8)
C12	C11	C10	108.0(4)	O4	C39	C66B	108.1(7)
C11	C12	C13	105.2(3)	O4	C39	C67A	120.7(14)
C12	C13	C14	102.5(3)	C67A	C39	C66A	107.9(15)
C19	C13	C12	113.2(4)	C67B	C39	O4	119.3(15)
C19	C13	C14	109.4(3)	C67B	C39	C66B	131.9(16)
C19	C13	C20	112.2(4)	O1A	C61A	C62A	109.3(11)
C20	C13	C12	107.1(4)	O2A	C61A	O1A	126.5(15)
C20	C13	C14	112.0(3)	O2A	C61A	C62A	124.1(14)
C10	C14	C13	102.1(3)	O1B	C61B	C62B	112.3(14)
C15	C14	C10	109.1(4)	O2B	C61B	O1B	123.4(16)
C15	C14	C13	112.8(4)	O2B	C61B	C62B	124.2(18)
C15	C14	C16	108.0(3)	C63A	C62A	C61A	125.3(15)
C16	C14	C10	116.4(4)	C63B	C62B	C61B	125.0(18)
C16	C14	C13	108.4(4)	C62A	C63A	C64A	123(2)
C14	C16	C17	113.2(4)	C62B	C63B	C64B	129.0(14)
C18	C17	C16	118.0(4)	C65A	C64A	C63A	123(2)

C17	C18	C19	117.1(4)	C68A	C64A	C63A	118.7(19)
C17	C18	C24	117.5(4)	C68A	C64A	C65A	118.4(18)
C24	C18	C19	117.8(4)	C65B	C64B	C63B	121.0(15)
C25	C18	C17	117.5(4)	C65B	C64B	C68B	119.1(18)
C25	C18	C19	115.0(4)	C68B	C64B	C63B	119.8(17)
C25	C18	C24	59.1(3)	C66A	C65A	C64A	120.6(16)
C13	C19	C18	112.2(3)	C66B	C65B	C64B	121.2(17)
C21	C19	C13	114.8(4)	O3A	C66A	C39	105.9(11)
C21	C19	C18	113.4(4)	O3A	C66A	C65A	127.6(14)
C22	C21	C19	111.4(4)	C65A	C66A	C39	126.2(13)
C21	C22	C23	110.1(4)	O3B	C66B	C39	123.9(11)
C22	C23	C24	112.9(4)	O3B	C66B	C65B	123.1(14)
C22	C23	C28	114.9(4)	C65B	C66B	C39	113.0(12)
C24	C23	C28	113.5(4)	C39	C67A	C68A	130(2)
C23	C24	C18	120.3(4)	C39	C67B	C68B	111(2)
C25	C24	C18	59.7(3)	C64A	C68A	C67A	117(2)
C25	C24	C23	121.7(4)	C67B	C68B	C64B	123(2)

Table S6. Hydrogen Atom Coordinates (Å×10^4) and Isotropic Displacement Parameters (Å^2×10^3) for 24MCA-FA.

**Atom**	***x***	***y***	***z***	**U(eq)**
H4	-7571.6	8103.4	-5287.45	147
H1A	-8123.55	-11596.87	-2726	142
H1B	-9260.11	-11478.98	-2886.72	142
H1C	-8463.11	-10266.83	-2825.27	142
H1BA	-6172.08	-10196.99	-2927.37	136
H1BB	-5653.87	-11175.22	-3094.84	136
H1BC	-5899.8	-11613	-2860.49	136
H2	-7546.85	-12347.51	-3057.02	93
H5A	-8719.47	-11112.69	-3504.18	120
H5B	-8424.05	-12376.35	-3366.63	120
H6A	-7115.92	-9092.49	-3185.66	87
H6B	-8573.15	-9038.67	-3132.27	87
H7A	-9086.29	-8980.34	-3481.98	82
H7B	-7662.67	-9196.82	-3546.66	82
H8	-7135.61	-7185.9	-3426.72	78
H9A	-9735.5	-6850.95	-3336.13	107
H9B	-8705.88	-5768.24	-3320.47	107
H9C	-8650.8	-6936.92	-3165.27	107
H10	-9115.1	-7254.7	-3727.37	72
H11A	-6541.76	-7857.94	-3770.68	84
H11B	-7718.95	-8477.7	-3886.73	84
H12A	-7286.14	-7282.65	-4161.23	84
H12B	-6241.79	-6523.74	-4032.31	84
H15A	-7002.39	-5025.34	-3512.9	106
H15B	-6822.08	-4163.52	-3714.09	106
H15C	-6200.19	-5524.42	-3705.87	106
H16A	-9126.33	-4396.52	-3565.81	75
H16B	-10017.4	-5209.26	-3712.91	75
H17A	-10121	-3712.24	-3944.87	74
H17B	-9481.46	-2824.12	-3776.77	74
H19	-6683.58	-4406.41	-4042.84	77
H20A	-9177.05	-6300.12	-4247.7	101
H20B	-9909.95	-5461.83	-4081.42	101
H20C	-9589.53	-6890.61	-4029.62	101

H21A	-6844.53	-5172.68	-4373.94	88
H21B	-8251.98	-4675.34	-4395.44	88
H22A	-6758.25	-3320.99	-4561.52	94
H22B	-6159.6	-3036.82	-4337.92	94
H23	-8662.32	-2512.73	-4456.7	73
H25A	-7682.02	-1675.49	-3849.38	86
H25B	-6571.99	-2259.65	-4003.5	86
H26A	-9741.14	-1267.13	-3982.97	84
H26B	-10209.65	-1697.26	-4208.54	84
H27A	-9970.58	503.49	-4211.78	99
H27B	-8561.08	386.25	-4129.21	99
H29A	-6527.23	-1494.33	-4721.89	128
H29B	-7224.92	-188	-4758.29	128
H29C	-7997.8	-1468.74	-4768.3	128
H30A	-9423.05	-529.89	-4516.97	94
H30	-9426.07	-334.88	-4536.97	94
H41A	-6611.98	-261.79	-4209.69	162
H41B	-6250.13	455.15	-4419.63	162
H41C	-5710.26	-916.11	-4375.86	162
H62A	-10065.43	3391.16	-4776.4	86
H62B	-7505.48	3009.89	-4665.31	85
H63A	-7591.31	2857.04	-4689.43	82
H63B	-9818.55	3630.15	-4838.17	76
H65A	-9767.69	4911.91	-4970	78
H65B	-9996.27	5384.4	-5074.69	78
H67A	-5659.14	6200.34	-5025.94	113
H67B	-5730.8	6392.06	-4964.1	113
H68A	-6068.01	4432.31	-4826.28	98
H68B	-6674.26	4656.22	-4794.53	98
H69A	-10992.27	6415.64	-5329.3	193
H69B	-11114.38	7420.72	-5146.06	193
H69C	-10793.2	5993.9	-5092.92	193
H69D	-11065.7	7685.17	-5204.46	193
H69E	-10749.57	6316.68	-5290.57	193
H69F	-10780.86	7483.39	-5446.37	193

Table S7. Atomic Occupancy for 24MCA-FA.

**Atom**	***Occupancy***	**Atom**	***Occupancy***	**Atom**	***Occupancy***
O1A	0.5	O1B	0.5	O2A	0.5
O2B	0.5	O3A	0.5	O3B	0.5
H30A	0.5	H30	0.5	C61A	0.5
C61B	0.5	C62A	0.5	H62A	0.5
C62B	0.5	H62B	0.5	C63A	0.5
H63A	0.5	C63B	0.5	H63B	0.5
C64A	0.5	C64B	0.5	C65A	0.5
H65A	0.5	C65B	0.5	H65B	0.5
C66A	0.5	C66B	0.5	C67A	0.5
H67A	0.5	C67B	0.5	H67B	0.5
C68A	0.5	H68A	0.5	C68B	0.5
H68B	0.5	C69A	0.5	H69A	0.5
H69B	0.5	H69C	0.5	C69B	0.5
H69D	0.5	H69E	0.5	H69F	0.5

Supplementary Information 5

The fragmentation pattern of MS/MS analysis and the data of NMR analysis of compound-X.

A. **Fragmentation pattern of MS/MS analysis**
B. **^{1}H NMR (600 MHz, $CDCl_3$)**
C. **^{13}C NMR (150 MHz, $CDCl_3$)**
D. **COSY ($CDCl_3$)**
E. **HSQC ($CDCl_3$)**
F. **HMBC ($CDCl_3$)**
G. **NOESY ($CDCl_3$)**

A. Fragmentation pattern of MS/MS analysis

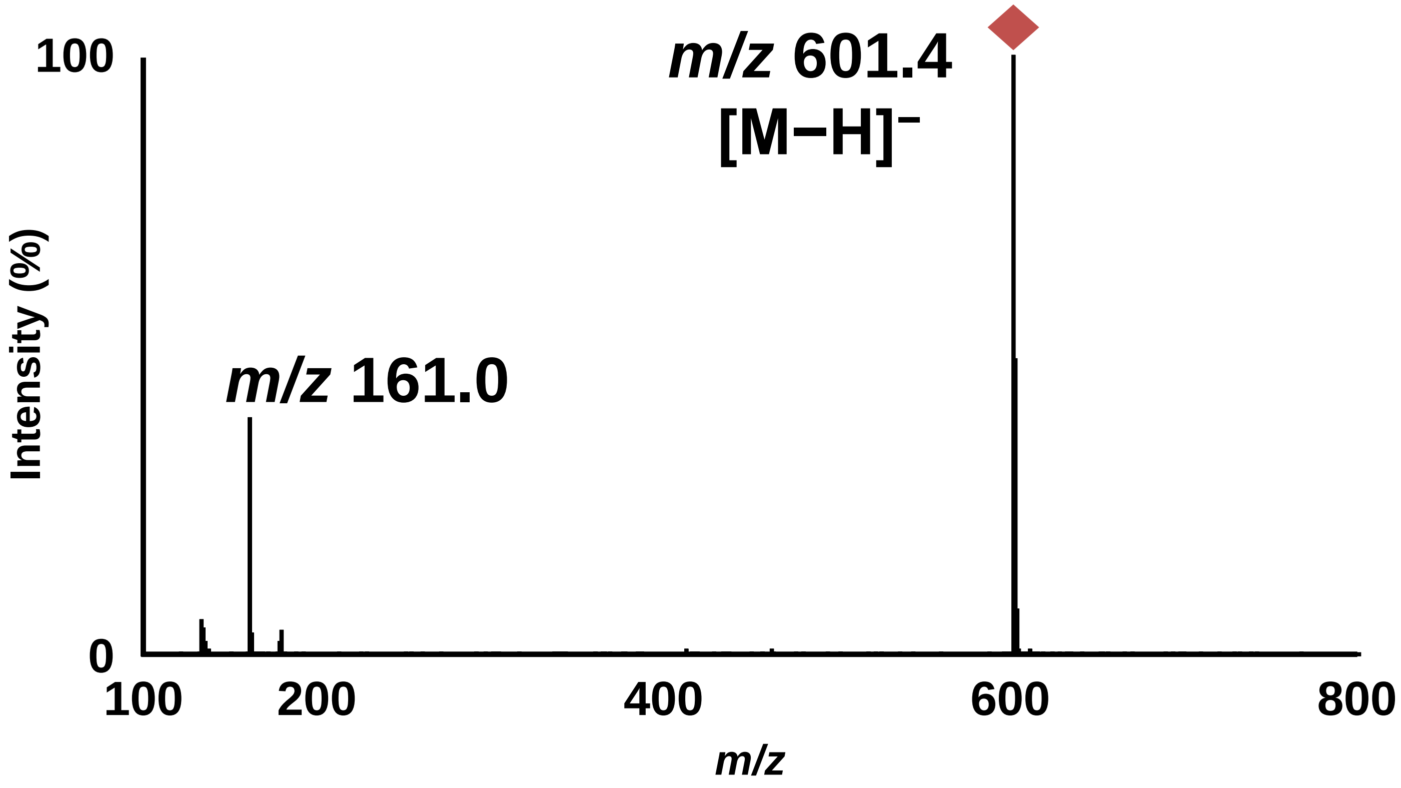

B. ^1H NMR (600 MHz, $CDCl_3$)

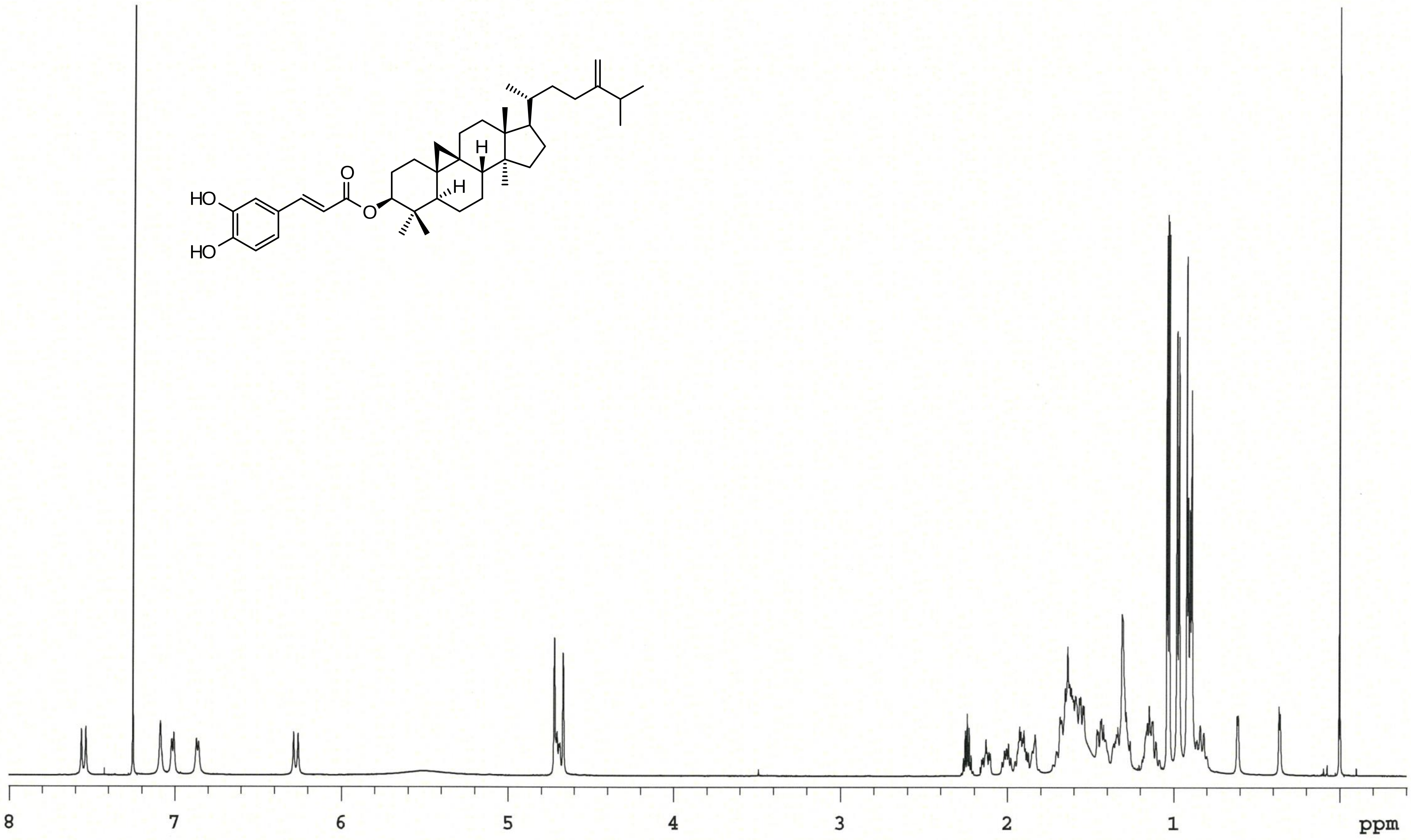

C. ^{13}C NMR (150 MHz, $CDCl_3$)

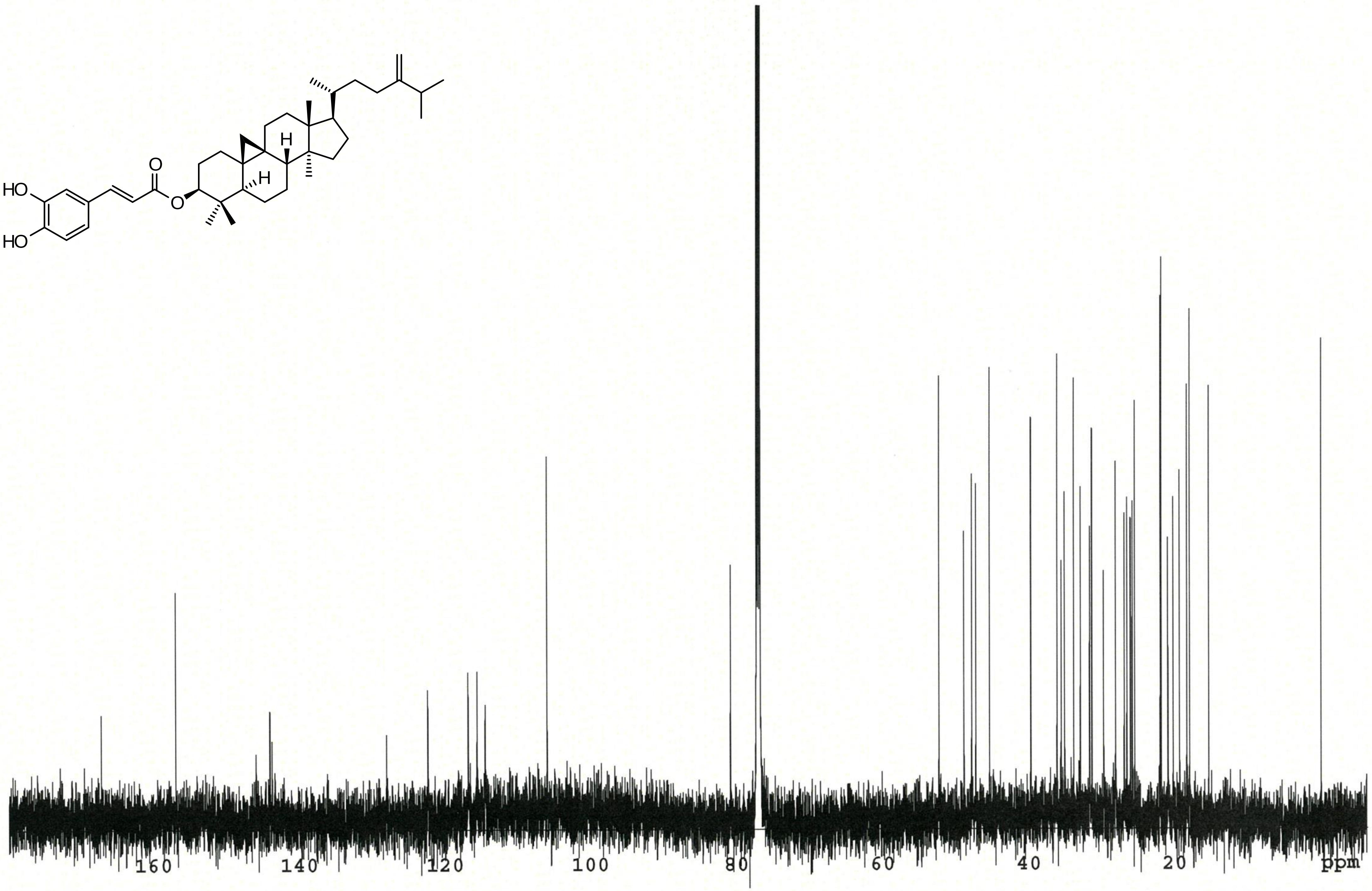

D. COSY ($CDCl_3$)

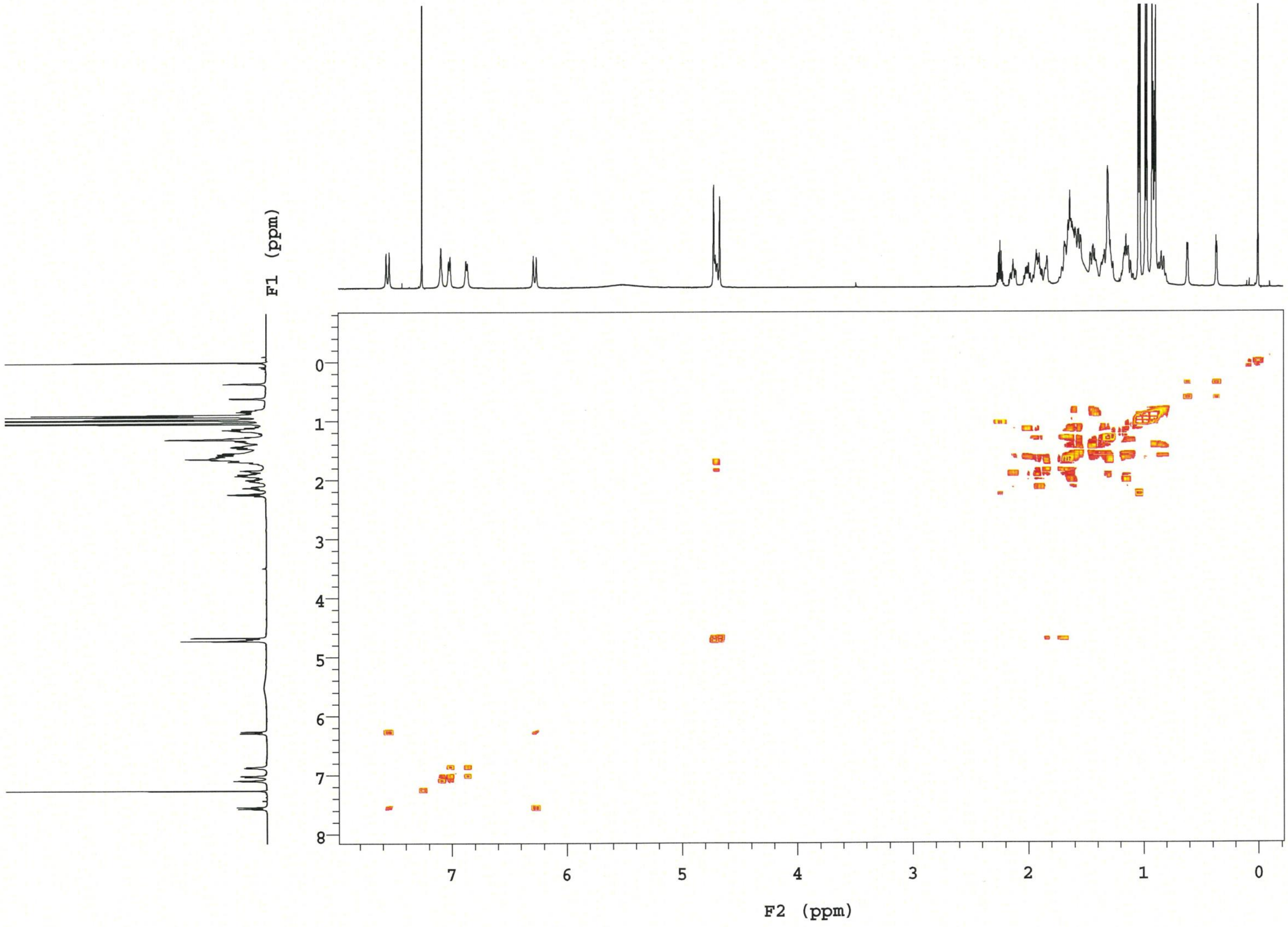

E. HSQC ($CDCl_3$)

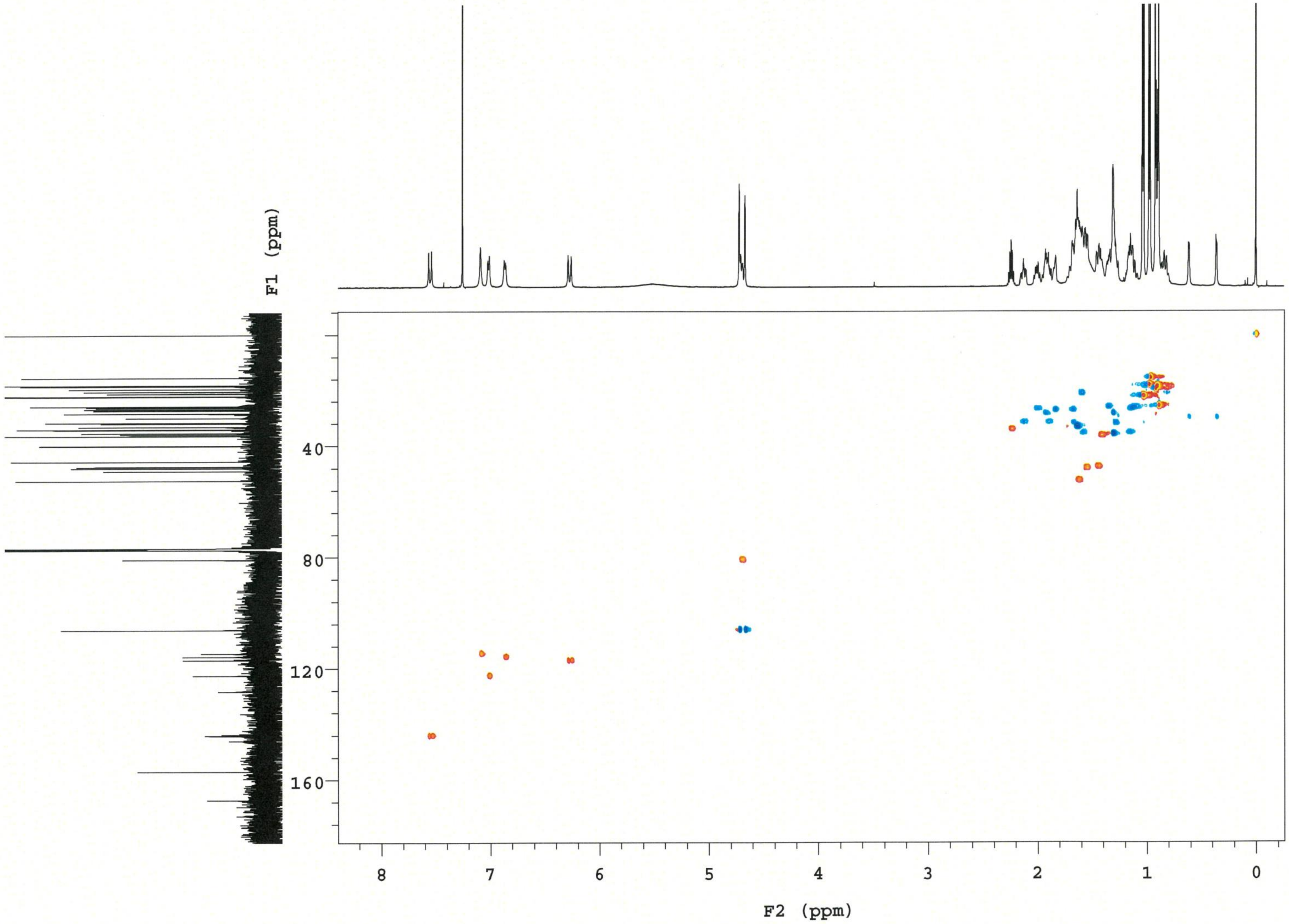

F. HMBC ($CDCl_3$)

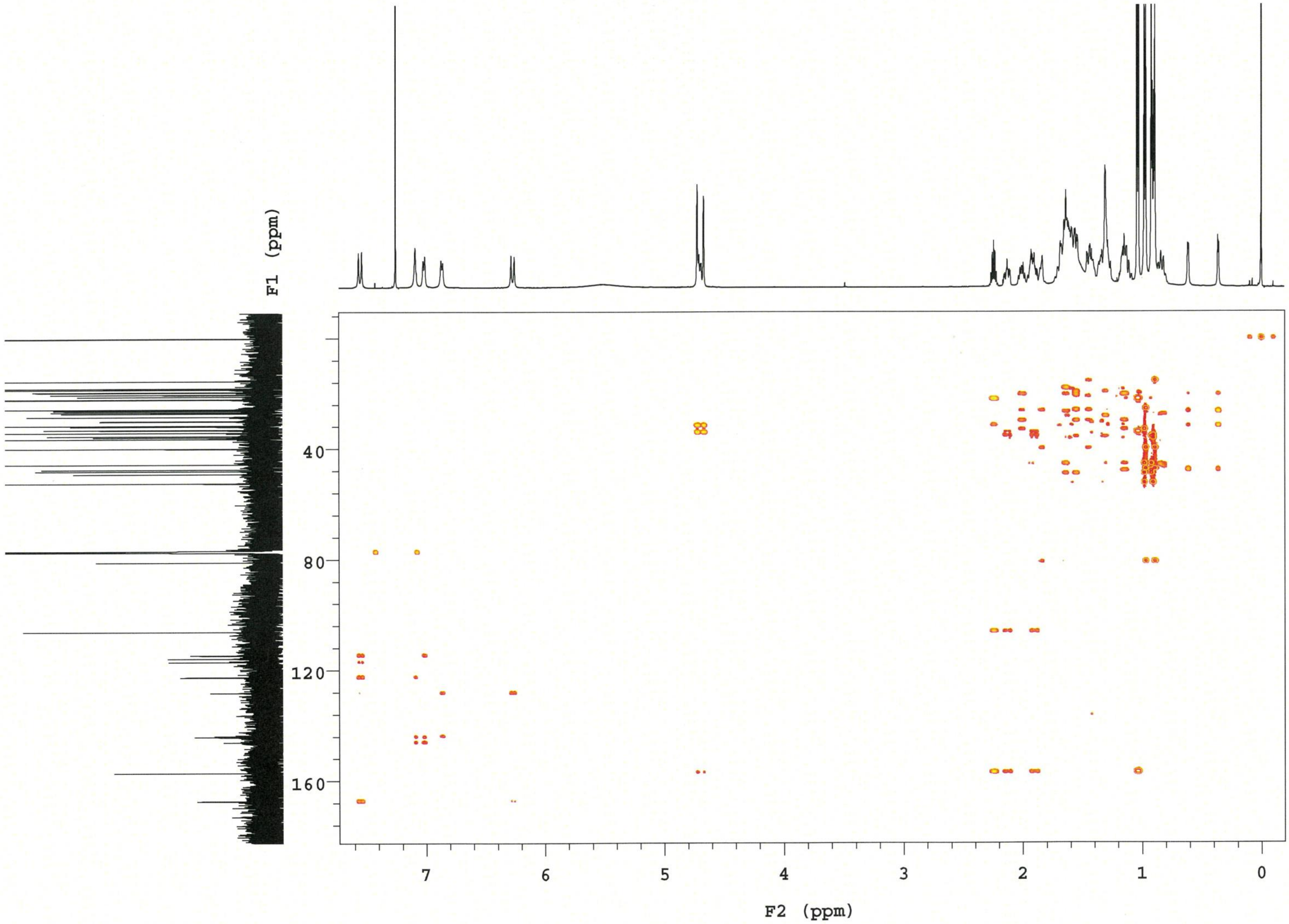

G. NOESY ($CDCl_3$)

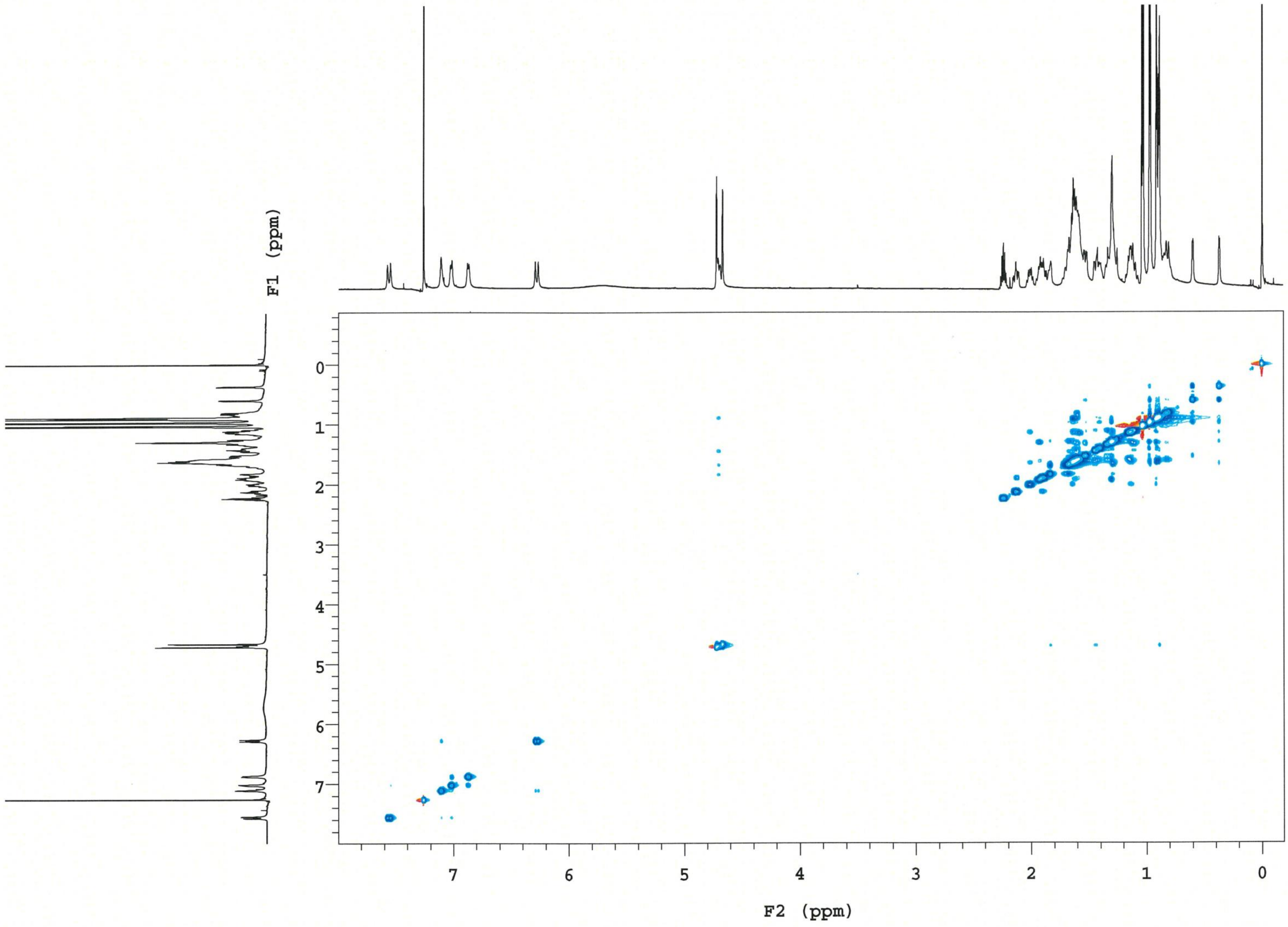

Supplement: Supplementary file 1 — Supplementary Information [file 41598_2019_48985_MOESM1_ESM.pdf]
